# ABT-263, a BCL-2 inhibitor, selectively eliminates latently HIV-1-infected cells without viral reactivation

**DOI:** 10.1371/journal.pone.0322962

**Published:** 2025-05-20

**Authors:** Jeong Eun Kang, Hyun Wook Seo, Dong-Eun Kim, Young Hyun Shin, Songmee Bae, Cheol-Hee Yoon

**Affiliations:** Division of Chronic Viral Disease Research, Center for Emerging Virus Research, National Institute of Health, Cheongju, Republic of Korea; University of Pennsylvania, UNITED STATES OF AMERICA

## Abstract

Human immunodeficiency virus-1 (HIV-1) is a hazardous pathogen responsible for causing acquired immunodeficiency syndrome (AIDS). HIV-1 provirus survives in latently infected cells for a long time, despite treatment with combinational anti-retroviral therapy (cART); therefore, it is considered as a major obstacle in HIV-1 treatment. Several strategies have been developed to selectively eliminate latently HIV-1-infected cells; however, clinical success has not yet been reported. Here, we identified several key factors associated with cell apoptosis, which were upregulated in latently infected cells. Subsequently, we screened compounds targeting these factors to selectively kill latently HIV-1-infected cells. Among these, ABT-263 (Navitoclax), a BCL-2 inhibitor, exhibited a potent and selective killing effect on latently HIV-1-infected cells and exerted synergistic effects with combinations of other compounds targeting myeloid cell leukemia-1 (MCL-1), X-linked inhibitor of apoptosis protein (XIAP), and BAX. In an *ex vivo* model, latently HIV-1-infected memory CD4^+^ T cells were efficiently eliminated via treatment with ABT-263 alone and its combinations with other modulatory compounds. Taken together, our results demonstrate that the balance of pro- and anti-apoptotic factors is crucial for the survival of latently HIV-1-infected cells. Thus, disrupting this balance using ABT-263 or combinations having ABT-263 without proviral reactivation may be useful for developing a novel strategy to eliminate latently infected cells in individuals infected with HIV-1.

## Introduction

Acquired immune deficiency syndrome (AIDS) caused by human immunodeficiency virus-1 (HIV-1) infection poses a global challenge; the number of person infected with HIV-1 is estimated approximately 38 million globally in 2024 [1,2]. It belongs to the *Retroviridae* family and carries two copies of single-stranded RNA genome, which is enveloped in a lipid bilayer spiked with gp120 and gp41 glycoproteins [3]. After infection, the HIV-1 genome is converted to viral complementary DNA (cDNA) using its reverse transcriptase, which is then integrated into the host chromosome, followed by the establishment of proviral reservoirs latently infected with HIV-1 [3,4]. Highly active anti-retroviral therapy efficiently suppresses viral replication, thereby hindering AIDS progression [5]. However, the latently HIV-1-infected cells are not completely eliminated, posing a major obstacle for developing an efficient HIV-1 cure [6].

Apoptosis is an important cellular regulatory mechanism that leads to the death of cells that are damaged or unnecessary for maintaining systemic homeostasis [7,8]. The apoptotic pathway is distinguished by the extrinsic and intrinsic pathways. The extrinsic pathway is activated in response to the binding of ligands, such as CD95 or tumor necrosis factor-alpha (TNF-α), to their receptors, followed by the formation of death-inducing signal complex (DISC), which activates caspase-8 and subsequently a series of effector caspases [9]. The intrinsic pathway is activated by several stimuli, such as pathogen infection, genotoxic cancer drugs, hypoxia, and growth factor depletion [7]. These stimuli facilitate the release of cytochrome C and second mitochondria-derived activator of caspase/direct inhibitor of apoptosis-binding proteins (SMAC/DIABLO) from the mitochondrial outer membrane, which activates caspase-9 and subsequent serial effector caspases (caspase-3, -6, and -7). Caspase activity is negatively regulated by inhibitors of apoptosis (IAPs), including an X-linked inhibitor of apoptosis protein (XIAP), cellular IAP-1/2 (c-IAP1/2), and survivin [7,9]. Mature SMAC/DIABLO negatively regulates IAPs through interaction with its N-terminal motif, resulting in caspase activation and apoptosis [10]. However, HIV-1-infected cells maintain their cell survival status with manipulation of diverse signal pathways, even though they contain parasitic genetic materials unnecessary for the cells [11,12]. Several apoptosis regulators, including B-cell leukemia/lymphoma 2 protein (BCL-2)-associated X (BAX) [13], IAP [10], and baculoviral IAP repeat containing 2 (BIRC2) [14,15], are differentially expressed in latently HIV-1-infected cells [13], which might be involved in the regulation of cellular apoptosis. Indeed, autophagic flux and interactions with immune cells contribute to HIV-1 latency [16,17].

Several studies have attempted to eliminate latently HIV-1-infected cells by manipulating cellular apoptotic pathways [14,17,18], where the number of latently HIV-1-infected cells (HIV-1 reservoirs) was reduced by treatment with a combination of an autophagy inhibitor (SAR-405) and an apoptosis inducer (BCL-2 inhibitor: ABT-263) under ingenol-3,20-dibenzoate (IDB)-stimulated latency reversal [17], a combination of a latency-reversing agent with SMAC/DIABLO mimetics, or SMAC/DIABLO mimetics alone [19]. In addition, genotoxins, such as etoposide (ES), selectively kill latently HIV-1-infected cells by sensitizing the p53-mediated apoptosis pathway without viral reversal in cell line models [13]. However, no drug is currently available for clinical use to eradicate latently HIV-1-infected cells because the unique intrinsic apoptosis pathway associated with latently HIV-1-infected cells has not been fully characterized [5,6].

In this study, we compared the protein expression signature of latently HIV-1-infected cells and uninfected parent cells and identified several cellular apoptosis regulators (p53, BAX, BCL-2, XIAP, and MCL-1) that were differentially expressed in latently HIV-1-infected cells. The agents capable of selectively killing the latently infected cells by targeting apoptosis regulators were selected. In addition, several combinations of agents that selectively killed the latently HIV-1-infected cells were evaluated, and their molecular mechanisms underlying apoptotic cell death were revealed. Taken together, our results may provide important information for understanding the biology of HIV-1 reservoir as well as developing novel strategies to selectively eliminate the latently HIV-1-infected reservoir.

## Materials and methods

### Cell lines, virus, and agents

HIV-1 non-infected parent cells (A3.01, Jurkat, and U937) and latently infected cells (ACH2, J1.1, and U1) were provided by the National Institute of Health (NIH) HIV Reagent Program (Bethesda, MD, USA). Cells were cultured in RPMI (Gibco, cat# 22400–089) with 1% penicillin streptomycin, 1% L-glutamine, and 10% heat inactivated fetal bovine serum (FBS, Gibco, cat# 10082–147) and incubated at 37 °C and 5% CO_2_. The used apoptosis-inducing agents and antibodies are listed in S1 Table. The apoptosis-inducing agents tested here were chosen for their commercial availability, efficacy, well-determined mode of action, and novelty in the HIV-1 research field. HIV-1 particles were prepared as previously described [13]. In brief, CXCR4-tropic molecular HIV-1 clone (pNL4–3) was transfected into HEK293T cells. Three days later, virus-containing cell culture supernatants were collected and filtered through a 0.45-μm membrane filter (Millipore Sigma, Burlington, MA, USA), aliquoted, and frozen at −80 °C for future experiments. Viral titration was performed as previously described [20]. In brief, 1 × 10^4^ of TZM-bl cells cultured in 96-well plates were infected with virus-containing medium diluted serially, in triplicate. Two days later, infectious viral particles were titrated using the beta-Galactosidase staining kit (Mirus Bio, Madison, WI, USA), and used to determine the multiplicity of infection (MOI) in each infection experiment.

### Cell viability assay

*In vitro* cytotoxicity was determined using the 3-(4,5-dimethylthiazol-2-yl)-2,5-diphenyltetrazolium bromide (MTT) assay kit (Abcam, cat# ab211091), as previously described [21]. Briefly, 1 × 10^5^ A3.01 and latently HIV-1-infected ACH2 cells cultured in 96-well plates were treated with variable concentrations (0–5 µ M) of apoptosis inducing agents (ABT-263, BTSA1, GX15–070, and Birinapant [BP]). At 24 h after drug treatment, cells were incubated with MTT solution for 3 h. Formed formazan crystals were dissolved in 10% SDS in 10 mM HCl; optical densities were then measured using a micro photometer reader (Biotek, VT, USA) at 595 nm. Data are presented as mean ± standard deviation (SD) (*n* = 2) compared with dimethyl sulfoxide (DMSO) control.

### Western-blotting analysis

One × 10^6^ A3.01 and latently HIV-1-infected ACH2 cells were treated with apoptosis inducing agents (BAX agonists, BCL-2 antagonists, and IAP antagonists) at different concentrations (0–5 µ M). At 24 h after treatment, total proteins were extracted from cells using cell lysis buffer containing a complete EDTA-free protease and phosphatase inhibitor cocktail. Western blotting analysis was performed as previously described [13]. Briefly, protein extracts were subjected to 12% sodium dodecyl sulfate polyacrylamide gel electrophoresis (SDS-PAGE) and transferred onto polyvinylidene difluoride (PVDF) membranes. The membranes were incubated with appropriate antibodies and visualized using chemiluminescence (Millipore, cat# WBKL S0500) according to the manufacturer’s protocols.

### Quantitative real-time reverse transcription polymerase chain reaction (RT-qPCR)

The mRNA expression levels of apoptosis-regulating genes in A3.01 and ACH2 cells were determined using RT-qPCR. RNA was extracted using the QIAamp Viral RNA Kit (Qiagen, cat# 52904) according to the manufacturer’s protocol. cDNA was synthesized using the First Strand Synthesis System Kit (Invitrogen, cat# BRL-18080–051) and an RNase inhibitor. cDNA templates (1 µl) were added to 10 µl of SYBR green master mix (Applied Biosystems, cat# 4386708) and 0.5 pmol/µl of primer sets corresponding to each apoptosis regulating factor (p53, NOXA, PUMA, BAX, BCL-2, and XIAP) (Table 1) to a final volume of 20 µl adjusted with distilled water. RT-qPCR was performed according to the manufacturer’s protocol using the Quant Studio 3 real-time system (Applied Biosystems, CA, USA). The relative gene expression was calculated as follows using the 2^-Δ ΔCt^ method, where the fold change of enrichment = 2 delta ((Ct-target)-(Ct-reference)). The ΔCt values were determined using Quant Studio PCR system software (Applied Biosystems) using the housekeeping gene GAPDH as an endogenous control. The expression of each gene was normalized to that of GAPDH in each sample.

**Table 1:** Sequences of quantitative real-time polymerase chain reaction (RT-qPCR) primers.

Gene	Forward primer	Reverse primer
P53	5’-GGCCCACTTCACCGTACTAA-3’	5’-GTGGTTTCAAGGCCAGATGT-3’
NOXA	5’-CTTGGAAACGGAAGATGGAA-3’	5’-CGCCCAGTCTAATCACAGGT-3’
PUMA	5’-GCCCAGACTGTGAATCCTGT-3’	5’-TCCTCCCTCTTCCGAGATTT-3’
BAX	5’-TTTGCTTCAGGGTTTCATCC-3’	5’-CAGTTGAAGTTGCCGTCAGA-3’
BCL-2	5’-GGATGCCTTTGTGGAACTGT-3’	5’-AGCCTGCAGCTTTGTTTCAT-3’
XIAP	5’-GCGGTTCAGTTTCAAGGACA-3’	5’-CGCCTTAGCTGCTCTTCAGT-3’
GAPDH	5’-GAGTCAACGGATTTGGTCGT-3’	5’-TTGATTTTGGAGGGATCTCG-3’

### Flow cytometry analysis

Populations of early apoptotic (annexin V-FITC-positive, propidium iodide (PI)-negative) and late apoptotic (annexin V-FITC-positive, PI-positive) cells were analyzed using flow cytometry with anti-annexin V-FITC and PI staining [13]. Briefly, 1.5 × 10^5^ cells were treated with 1 μM of apoptosis inducing agents (BAX agonists, BCL-2 antagonists, and IAP antagonists) for 24 h, then incubated with anti-annexin V-FITC antibody (Abcam, cat# ab10485) and PI (Invitrogen, cat# R37169) for 10 min at 24 °C. The stained cells were detected using flow cytometry (BD FACS Lyric, NJ, USA) and analyzed using BD FACSuite software (NJ, USA). The sub-G1 cell populations were analyzed as previously described [13]. Briefly, cells were incubated with drugs for 24 h and then fixed with 70% ice-cold ethanol and incubated overnight at 4 °C. The fixed cells were incubated with 100 µ g/ml RNase A and 50 µ g/ml PI solution at 37 °C for 1 h. To measure the intracellular DNA content, at least 10,000 events were analyzed.

### Cytochrome C release analysis

Three × 10^6^ A3.01 and latently HIV-1-infected ACH2 cells were treated with 1 µ M apoptosis inducing agents (ABT-263, BTSA1, and GX15–070) for 12 h. After treatment, the mitochondria and cytosol were isolated from the cells using a mitochondria/cytosol fractionation kit (Abcam, cat# ab65320) according to the manufacturer’s protocol. Cytochrome C released from the cytosolic and mitochondrial fractions was detected using Western blotting analysis.

### Mitochondrial membrane potential analysis (ΔΨ)

Mitochondrial membrane potential analysis was performed as previously described [22,23]. Briefly, 1 × 10^6^ A3.01 and ACH2 cells were treated with 1 µ M apoptosis inducing agents (ABT-263, BTSA1, GX15–070, and Birinapant) for 24 h. The cells were then stained with JC-1 dye (5′,6′,6′-tetrachloro-1,1′,3,3′-tetrathylbenzimidazolylcarbocyanine iodide) (Invitrogen, cat# T3168) at 37 °C for 30 min according to the manufacturer’s instructions. Relative degrees of mitochondrial polarization were quantified by measuring the red-shifted JC-1 aggregates on FL-2 (PE, high zeta potentials (ΔΨ)) channel and the green-shifted monomers on FL-1 (FITC, low ΔΨ) channel using flow cytometry (BD FACS lyric, NJ, USA), and visualized using fluorescence microscopy (Olympus IX-83, Shinjuku, Japan).

### Acute and primary HIV-1 infection experiments

One × 10^6^ A3.01 cells were acutely infected with HIV-1_NL4–3_ at an MOI of 0.1, and subsequently cultured for 24 h. The protein levels in the cells were analyzed by Western blotting using the appropriate antibodies. The acutely infected A3.01 cells were treated with 1 μM apoptosis inducing agents (ABT-263, BTSA1, GX15–070, and Birinapant) for 24 h. After treatment, the sub-G1 population of acutely infected A3.01 cells was analyzed by flow cytometry (BD FACS lyric, NJ, USA) using PI staining [24].

Primary HIV-1-infected cells were established as previously described [25,26]. Naïve CD4^+^ T cells were isolated from 1 × 10^8^ cells of commercially available human PBMCs (Stemcell, cat# ST70025) by magnetically negative sorting using the Easysep Human Naïve CD4^+^ T Cell Isolation Kit (Stemcell, cat# 17555). Briefly, the human PBMCs contained into 5 ml round-bottom tube were incubated with 50 μl of antibody cocktail at 24 °C for 5 min and further incubated with 50 µl of Rapidshere under the same condition. Sequentially, the cells were placed into the Easysep magnet (Stemcell, cat# 18000) and incubated for 3 min. The cells-containing supernatant was transferred to a new 5 ml round-bottom tube and the tube was placed into magnet for a second round separation. The enriched naïve CD4^+^ T cells were collected by careful pipetting and analyzed by flow cytometry (BD FACS lyric, NJ, USA). The naïve CD4^+^ T cells were cultured in a 100 mm cell culture dish with 125 µl anti-CD3/CD28 T cell activating antibodies (Stemcell, cat# 10971) in a 5% CO_2_ incubator at 37 °C for 72 h. The cells were washed and then cultured with 10 µg/ml IL-4 antibody (R&D system, cat# MAB204), 20 µg/ml IL-12 antibody (R&D system, cat# AF-219-NA), 4 µg/ml recombinant TGF-β1 (R&D system, cat# 7754-BH-025), and 30 U/1 × 10^6^ cells of human IL-2 (Roche, cat# 10799068001) for 96 h. The differentiated CD4^+^ T cells (including central memory (Tcm), effector memory (Tem), transitional memory (Ttm) and effector (Teff) CD4^+^ T cells etc.) were analyzed by flow cytometry (BD FACS Lyric) using fluorescent labeled CD4, CD45RA, CD45RO, CCR7, and CD27 antibodies as previously described [25,26].

After analysis of the differentiated cell populations, the cells were infected with HIV-1_NL4–3_ at an MOI of 1 by spinoculation for 2 h at 300 g and incubated for 7 days under IL-2 treatment. Then, the virus-infected cells were treated with apoptosis inducing agents (0.05 µ M ABT-263, 0.5 µ M BTSA-1, and 0.5 µ M GX15–070). After 24 h, populations of HIV-1 p24 positive and apoptotic cells were analyzed upon the addition of anti-CD3/CD28 T cell activator by flow cytometry (BD FACS lyric) using annexin V-FITC/PI-staining.

### Chip-seq data analysis

To analyze histone modifications for activation markers (H3K4me3 and H3K9ac) in A3.01 and latently HIV-1-infected ACH2 cells, Chip-seq data of these cells were obtained from the NCBI Gene Expression Omnibus (GEO) data base (GSM1404656: A3.01 H3K9ac, GSM1404657: ACH2 H3K9ac, GSM1404660: A3.01 H3K4me3, and GSM1404661: ACH2 H3K4me3) [27]. Chip-seq leads were aligned to the GRCh 38 (Genome Reference Consortium Human Reference 38 assembled from the human genome released by the UCSC, https://genome.ucsc.edu) (NCBI: GCA 000001405.29). Histone modifications (H3K4me3 and H3K9ac) of apoptosis-regulating factors were analyzed in coding as well as non-coding regions of BAX (NM 138761, chr19:20,772 bp), BCL-2 (NM 000633, chr18:589,272 bp) and XIAP (NM 001167, chrX: 17,973 bp).

### Statistical analysis

All data and graphs are presented as means and SDs of three independent experiments. Data were compared using Student’s *t* test, and a p-value < 0.05 was considered statistically significant.

## Results

### Different expression pattern of apoptosis-related factors in latently HIV-1-infected cells

**Fig 1:**
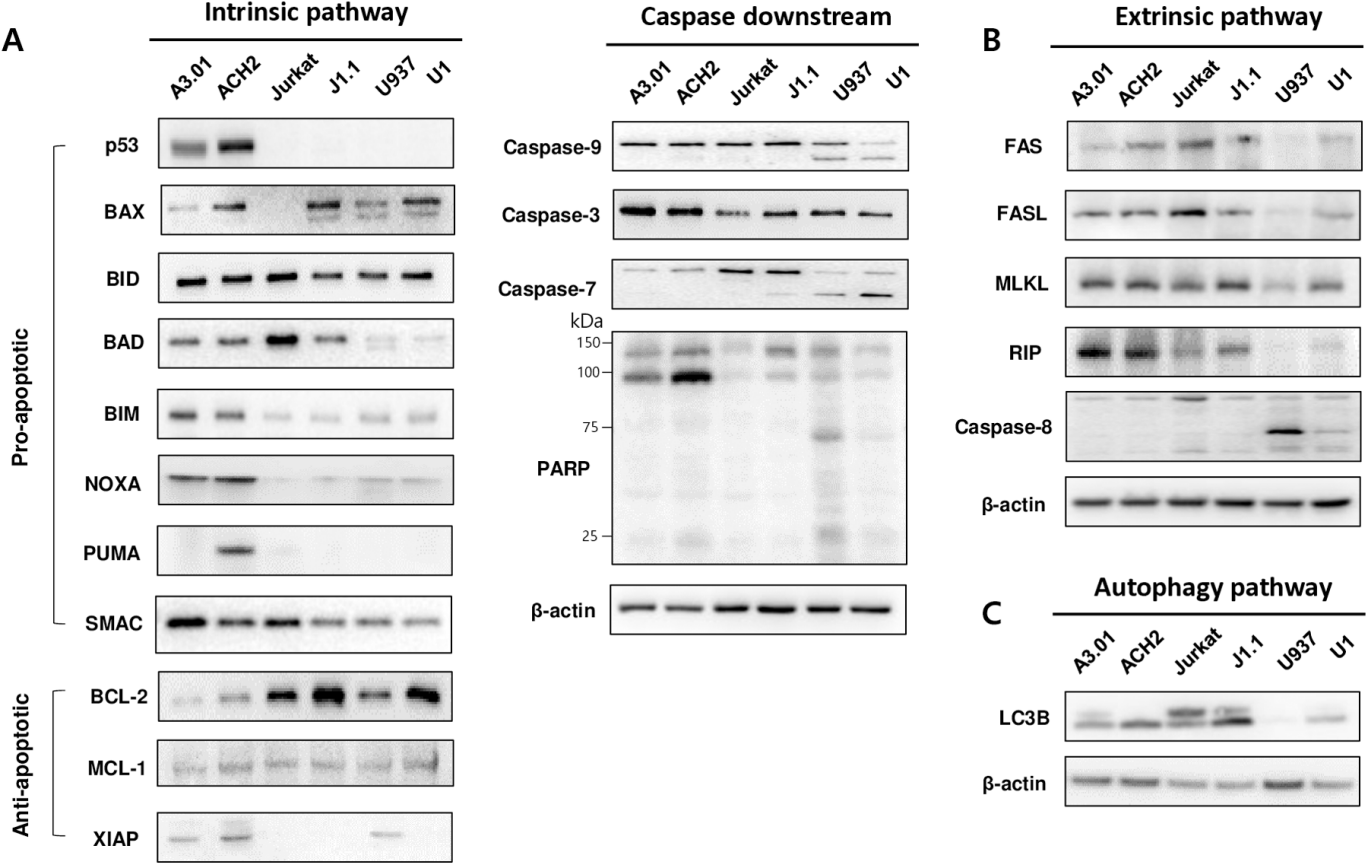
Differential expression of apoptosis regulating factors in latently HIV-1-infected cells. (A) The protein expression levels of pro-apoptotic and anti-apoptotic factors, caspase-3, -7, and -9; and PARP were compared between latently HIV-1-infected (ACH2, J1.1, and U1) cells and the parent cells (A3.01, Jurkat, and U937) by Western blotting analysis using the indicated antibodies. (B) Comparison of the protein expression levels of FAS, FASL, MLKL, RIP, and caspase-9 associated with the extrinsic apoptosis pathway in latently HIV-1-infected and non-infected cells. (C) The protein expression level of LC3B is related to the autophagy pathway in these cells. Housekeeping β-actin was used as a loading control.

To identify the modulators that play a key role in the survival and death of latently HIV-1-infected cells, the protein expression levels of pro- and anti-apoptotic factors were compared between latently HIV-1-infected cells (ACH2, J1.1, and U1) and non-infected parental cells (A3.01, Jurkat, and U937) (Fig 1). Several different patterns of protein expression in the intrinsic apoptosis and autophagy pathways were observed between the latently infected and non-infected cells (Figs 1A and 1C). The expression patterns of the tumor suppressor gene *p53* and its target genes, BAX, NOXA, and PUMA, were higher in ACH2 (p53 wild type) cells than those in A3.01 cells, while BAX was highly expressed even in latently infected p53-defective J1.1 and U1 cells. The SMAC expression pattern was slightly lower in all three HIV-1-infected cells compared to that in their parent cells. The expression levels of BCL-2 were higher in all three latently HIV-1-infected cells compared to those in their parent cells. Slight upregulation of XIAP was observed only in ACH2 cells. Despite the difference in the expression signature of apoptotic factors in each cell type, no differences in caspase and PARP activities were observed in latently HIV-1-infected cells compared to those in their parent cells without stimuli. LC3B slightly matured in all three latently infected cell lines, as previously described (Fig 1C) [28], but significant difference of autophagic phenotype was not observed between latently HIV-1-infected and non-infected parent cells by analysis of immunocytochemistry (S1 Fig). The increased mRNA levels of factors which showed higher protein levels in ACH2 cells compared with those in A3.01 cells were also observed in ACH2 cells (S2 Fig). These findings indicated that apoptotic and anti-apoptotic factors may be well balanced in latently HIV-1-infected cells to maintain cell survival. The p53 defective cells were excluded from subsequent experiments owing to their physiological non-relevance.

### BCL-2 antagonist ABT-263 selectively kills the latently HIV-1-infected cells

**Fig 2:**
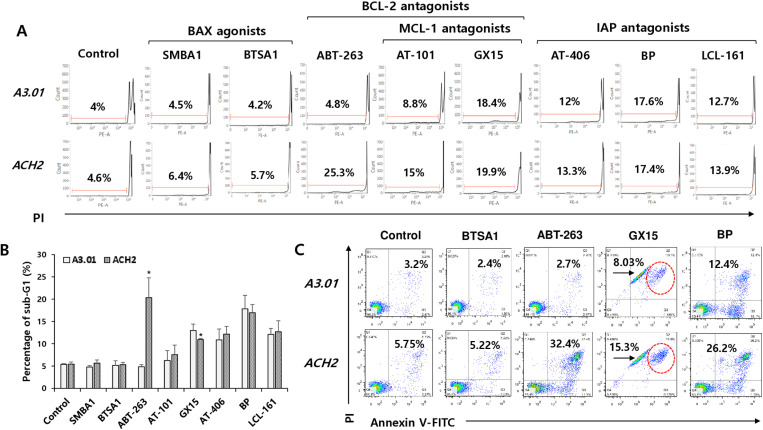
ABT-263 induced apoptosis of cells in latently HIV-1-infected cells. (A) The cells were treated with 1 μM BAX agonists (BTSA1 and SMBA1), BCL-2 antagonists (ABT-263, AT-101, and GX15-070), and IAP antagonists (AT-406, Birinapant [BP], and LCL-161) for 24 h. The sub-G1 population (dead cells) was determined by flow cytometry after PI staining. (B) Results of three independent sub-G1experients are represented as graphical data expressed as means ± SD (n = 3). *p < 0.05, compared with A3.01 cells treated with the same agent. (C) The cells were treated with indicated agents as described in (A). Early and late phases of apoptotic cells were analyzed by flow cytometry using annexin V-FITC/PI staining.

**Fig 3:**
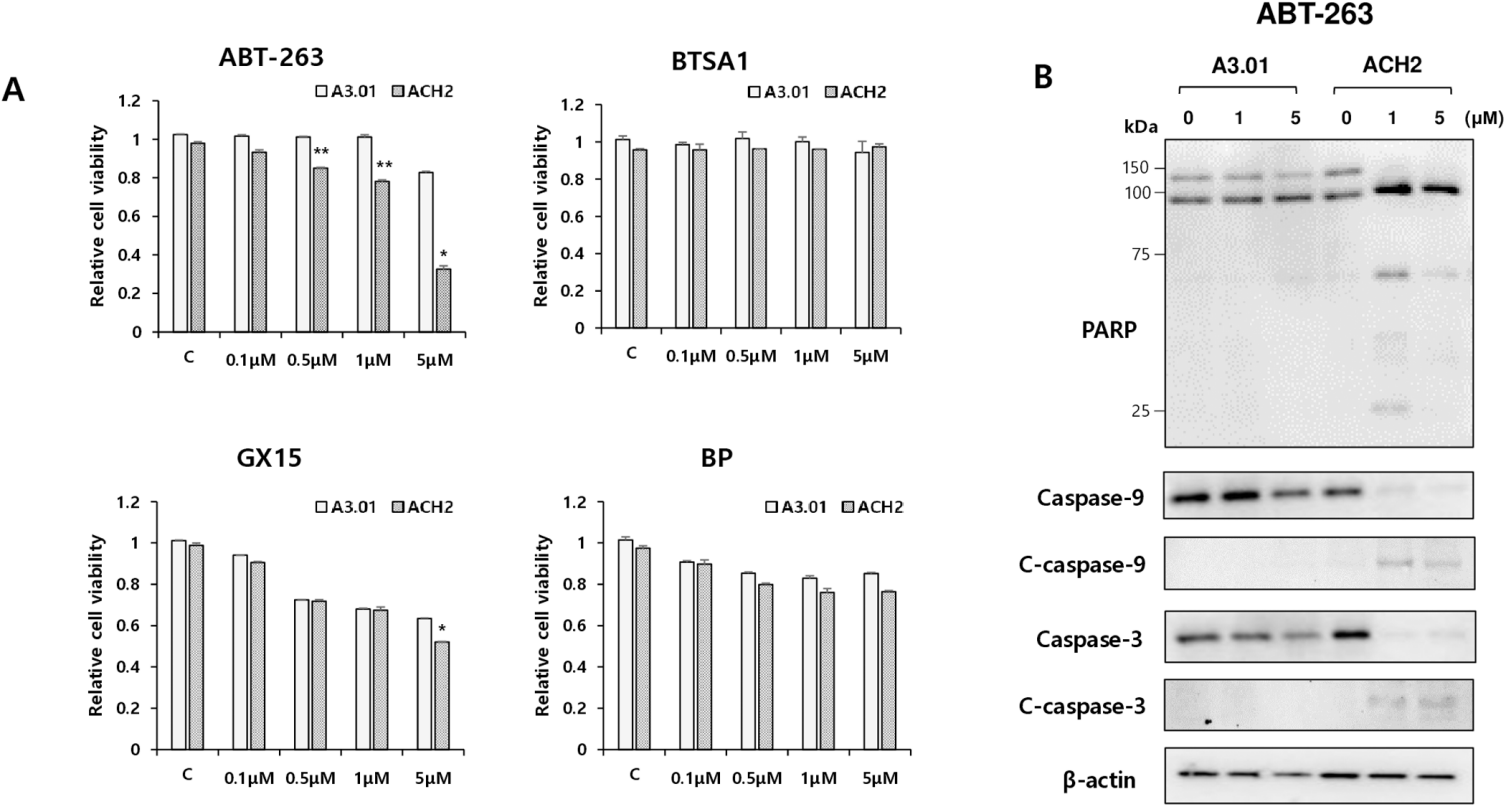
ABT-263 induced apoptosis executor proteins in latently HIV-1-infected cells. (A) A3.01 and ACH2 cells were treated with indicated apoptosis inducing agents at different concentrations (0.1, 0.5, 1, and 5 μM) for 24 h, and cell viabilities of these cells were determined using the MTT assay. Data are represented as the relative mean value ± SD (n = 3) compared with DMSO control. *p < 0.05, **p < 0.01, compared with A3.01 cells treated with the same conditions. (B) A3.01 and latently infected ACH2 cells treated with ABT-263 (0, 1, and 5 μΜ) for 24 h. At 24 h after treatment, levels of cleavage of PARP, caspase-3, and -9 were analyzed by Western blotting using the indicated antibodies. β-actin was used as a loading control.

To determine which agents targeting the differentially expressed factors could induce selective apoptotic cell death in latently HIV-1-infected cells, populations of apoptotic cells were analyzed in latently HIV-1-infected ACH2 and normal cells (A3.01) treated with BAX agonists (BTSA1 and SMBA1), BCL-2 antagonist (ABT-263), BCL-2/MCL-1 antagonists (AT-101 and GX15–070), and IAP antagonists (BP, AT-406, and LCL-161). Of these apoptosis-inducing agents, ABT-263 (Navitoclax) significantly induced a sub-G1 population of latently HIV-1-infected ACH2 cells, whereas other agents did not show a significant difference in the sub-G1 population between the two cell types under the experimental conditions (Figs 2A and 2B). The selective apoptotic effect of latently HIV-1-infected cells was observed in all four agents (BTSA1, ABT-263, GX15–070, and BP) by flow cytometric analysis using annexin V-FITC/PI staining, but the most selective potency was observed in ACH2 cells treated with ABT-263 (Fig 2C). In the cell viability assay based on MTT, the decrease in cell viability of latently HIV-1-infected ACH2 cells compared with that in the parent A3.01 cells was observed after treatment with ABT-263 in a dose-dependent manner (Fig 3A). In Western blotting analysis, cleaved apoptosis executors, PARP, and caspase-9 and -3 were only observed in latently HIV-1-infected ACH2 cells treated with ABT-263 (Fig 3B). These data revealed that the BCL-2 antagonist ABT-263 is the most potent and selective agent for killing latently HIV-1-infected cells in the absence of a latency reversal agent.

### Synergistic effect on selective apoptosis of latently HIV-1-infected cells

**Fig 4:**
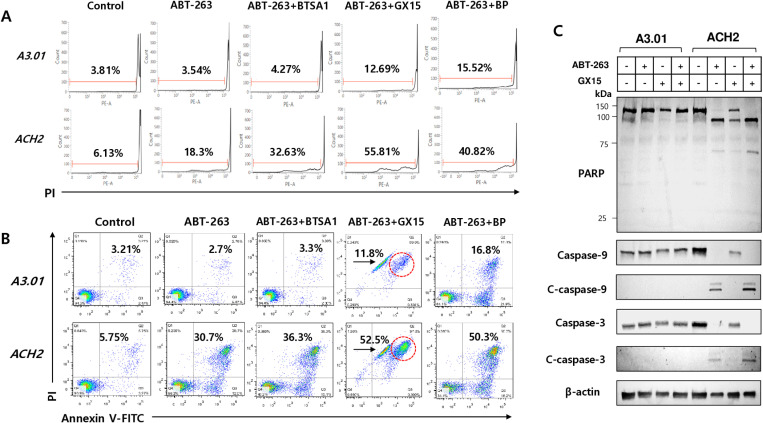
Synergistic effect of the combination of ABT-263 on the selective death of latently HIV-1-infected cells. (A) The cells were treated with ABT-263 (1 μM) or its combination with BTSA1 (1 μM), GX15-070 (1 μM), and BP (1 μM), respectively. At 24 h after treatment, the sub-G1 population of cells was determined by flow cytometry after PI staining. (B) The cells were treated with ABT-263 (1 μM) or its combinations (1 μM) for 24 h. Subsequently, early and late phase of apoptotic cells were analyzed by flow cytometry using annexin V-FITC/PI staining. (C) A3.01 and ACH2 cells were treated with ABT-263 (1 μM) and its combination with GX-15-070 (1 μM). At 24 h after treatment, expression of proteins was analyzed by Western blotting using the indicated antibodies and β-actin as a loading control.

To determine the optimal combination based on ABT-263 for the selective apoptosis of latently HIV-1-infected cells, the cells were treated with ABT-263 combined with other apoptosis-inducing agents. The sub-G1 population of latently infected ACH2 cells was markedly increased by treatment with ABT-263 combined with BTSA1, GX15–070, and BP compared with that observed with the treatment of ABT-263 alone (Fig 4A and S3 Fig). This synergism was also observed in the annexin V-FITC/PI staining assay (Fig 4B). ABT-263 combined with other agents (BTSA1, GX15–070, and BP) slightly increased the apoptotic cell death of non-infected A3.01 cells similar to that observed with each agent alone (Figs 2A–2C and 4). GX15–070 showed the strongest synergistic effect on the apoptosis of latently HIV-1-infected cells in combination with ABT-263 (Figs 4A and 4B). Synergism was also observed with cleavage-active forms of apoptosis executor proteins such as PARP, caspase-9, and -3 in latently HIV-1-infected cells treated with a combination of ABT-263 and GX15–070 (Fig 4C). ABT-263 combined with ES (a genotoxic agent inducing p53 activation) showed a weak synergistic apoptosis effect in ACH2 cells, as measured by flow cytometry (S4 Fig). These data indicated that the optimal selective apoptosis of latently HIV-1-infected cells might be associated with increased permeabilization of the mitochondrial outer membrane by BCL-2 inhibition and its combination with MCL-1 inhibition or BAX activation.

### Selective killing effect of latently HIV-1-infected cells associated with mitochondrial membrane permeabilization

**Fig 5:**
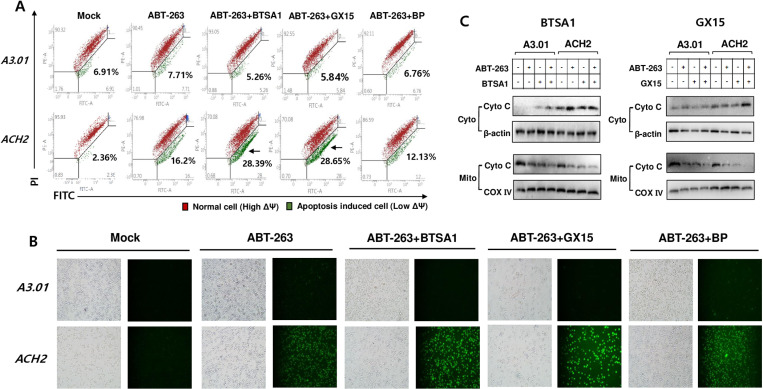
Change of mitochondrial membrane potential and cytochrome C release. (A) The cells were treated with ABT-263 (1 μM) or its combination with BTSA1 (1 μM), GX15-070 (1 μM), and BP (1 μM), respectively. At 24 h after treatment, the mitochondrial membrane potential of these cells was determined by flow cytometry after JC-1 staining. Red and green cells indicate high (PE) and low (FITC) mitochondrial membrane potential, respectively. (B) The cells were treated with ABT-263 and its combinations, and the cells containing low mitochondrial membrane potential (FITC) after staining with JC-1 were visualized using fluorescence microscopy. (C) The cells were treated with ABT-263 and its combinations for 12 h, and the mitochondria and cytosol were fractionated from those cells. The levels of cytochrome C were analyzed by Western blotting. COX IV and β-actin were used as loading controls. Cyto; Cytoplasm, Mito; Mitochondria.

To determine whether permeabilization of the mitochondrial outer membrane was selectively increased in latently HIV-1-infected cells treated with ABT-263 or its combination, the mitochondrial membrane potential was measured by flow cytometry analysis using JC-1 staining. Monomeric JC-1 (indicating a low mitochondrial membrane potential, green-colored cells) was exhibited at a higher level in the latently HIV-1-infected ACH2 cells compared with that in the non-infected cells under ABT-263 treatment. Its level increased more in ACH2 cells treated with ABT-263 combined with BTSA1 and GX15–070; however, a combination with BP inhibiting IAPs downstream of caspases did not exhibit this increase (Figs 5A and 5B). Treatment with ABT-263 alone and in combination with BTSA1 and GX15–070 increased the release of cytochrome C from the mitochondria of latently HIV-1-infected cells, which was consistent with the mitochondrial membrane potential determined by JC-1 staining (Fig 5C). The selective killing effects on latently HIV-1-infected cells have been previously examined under viral reactivation conditions. Therefore, we attempted to determine whether viral reactivation occurs during apoptosis of latently HIV-1-infected cells upon treatment with apoptosis-inducing agents. Treatment of a strong latency reversal agent, phorbol myristate acetate (PMA) exhibited the greatest viral reactivation, whereas GX15–017 showed slight reactivation. However, other agents did not exhibit significant viral reactivation (S5 Fig). These data revealed that ABT-263 and its combination can induce the selective killing of latently HIV-1-infected cells in the absence of viral reactivation.

### Apoptotic effect of ABT-263 on acute HIV-1 infection

**Fig 6:**
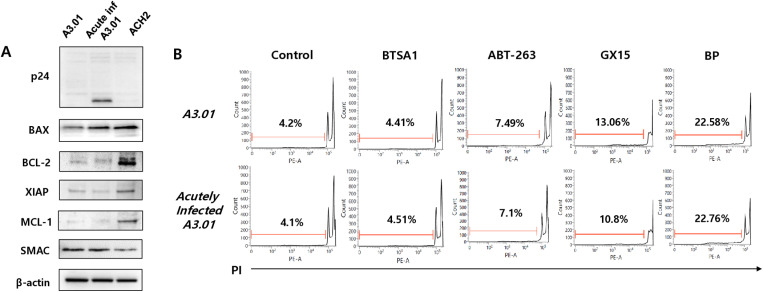
Apoptosis effects of apoptosis inducing agents on acutely HIV-1-infected cells. (A) A3.01 cells were infected with HIV-1_NL4-3_ at an MOI of 0.1. At 24 h after infection, the expression levels of proteins were measured by Western blotting using the indicated antibodies. (B) Cells infected acutely with HIV-1_NL4-3_ were treated with BTSA1 (1 μM), ABT-263 (1 μM), GX15-070 (1 μM), and BP (1 μM), respectively. At 24 h after treatment, the sub-G1 population of cells was determined by flow cytometry after PI staining.

Initially, the protein expression levels of apoptosis-regulating factors (BAX, BCL-2, XIAP, and MCL-1) were higher in the latently infected cells (ACH2) than those in A3.01 cells infected acutely with HIV-1 for 24 h. The acutely infected cells showed a slightly higher expression level of BAX compared to the uninfected cells, but the expression levels of BCL-2, MCL-1, XIAP, and SMAC did not change in the acutely infected cells (Fig 6A). Besides, ABT-263 and the other agents did not induce a sub-G1 population of the infected cells compared to the non-infected cells (Fig 6B). These data indicated that ABT-263 did not induce selective apoptosis in the acutely HIV-1-infected cells in contrast to the latently infected cells.

### Apoptotic effects of ABT-263 and its combinations on HIV-1-infected primary cell model

**Fig 7:**
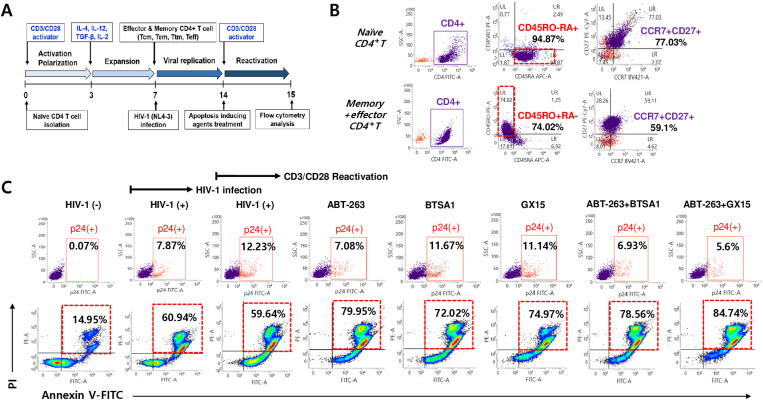
Selective killing effects of ABT-263 and its combination in a primary HIV-1 infection model. (A) Schematic process for establishment of primary HIV-1 infection model. The naïve CD4^+^ T cells sorted magnetically from human PBMCs were activated and expaned with treatment of anti-CD3/CD28 antibodies and cytokines (IL-4, IL-12, TGF-B1, and IL-2) for 7 days, that led to major differentiation of human memory and effector CD4^+^ T cells. After the differentiation, the cell mixtures were infected with HIV-1_NL4-3_ at an MOI of 1 for 7 days; the cells were then treated with apoptosis-inducing agents for 1 day. To determine the latently infected cells, the cells were reactivated with anti-CD3/CD28 antibodies and measured by flow cytometry. (B) The isolated naïve CD4^+^ T cells from human PBMCs (upper panel, CD45RO^-^RA^+^, 0 day) and differentiated CD4^+^ T cells (lower panel, CD45RO^+^RA^-^, 7 days) were analyzed by flow cytometry. (C) The differentiated CD4^+^ T cell mixture was infected with HIV-1_NL4-3_ for 7 days; the cells were then treated with ABT-263 (0.05 μM), BTSA1 (0.5 μM), GX15-070 (0.5 μM), and combinations of ABT-263 for 24 h and were subsequently reactivated with anti-CD3/CD28 antibodies. The populations of HIV-1 p24^+^ cells and early and late phases of apoptotic cells were analyzed by flow cytometry using HIV-1 p24 and annexin V-FITC/PI staining.

To determine the therapeutic effect of ABT-263 and its combinations in a primary cell model, effector and memory CD4^+^ T cells were differentiated from naïve CD4^+^ T cells by treatment with anti-CD3/CD28 antibodies and cytokines (IL-2, IL-12, IL-4, and TGF-β1) (Figs 7A and 7B). The differentiated cells were infected with HIV-1_NL4–3_ and treated with the indicated agents. The selective killing effects of the agents (ABT-263, BTSA1, and GX15–070) on the latently infected cells were analyzed, along with reactivation by treatment with anti-CD3/CD28 antibodies (Fig 7C). The productively infected cells were 7.87%, and reactivated cells were 12.23%, indicating that the latently infected cells were 4.35%. The population of total apoptotic cells was 60.94% in HIV-1-infected cells; the population was not increased by reactivation, which may be caused by massive cell death by abortive infection [29]. ABT-263 treatment increased the total apoptotic cell death and decreased the number of reactivated p24^+^ cells. These effects were minimized under treatment with BTSA1 or GX15–070 alone. Notably, the number of reactivated cells further decreased by treatment with ABT-263 combined with BTSA1 or GX15–070 compared to that observed by treatment with ABT-263 alone, thus increasing the total apoptotic cell population (Fig 7C). These results demonstrated that ABT-263 and its combination selectively eliminated the latently HIV-1-infected cells in the primary HIV-1 infection model.

## Discussion

During HIV-1 infection, latently HIV-1-infected provirus in memory CD4^+^ T cells contributes to the establishment of reservoir cells that play a role in infectious virus excretion, and are considered major obstacles to HIV-1 eradication. Eliminating HIV-1 reservoirs is indispensable for effective cure; however, selective markers for targeting the HIV-1 reservoir, as well as drugs capable of eradicating the reservoir have not yet been discovered.

HIV-1 infection regulates apoptosis in the infected cells through a variety of signaling pathways linked to pro- and anti-apoptotic factors [8,11]. The BCL-2 family, including pro-apoptotic (BAX, BAK, BAD, BID, and BIM) and anti-apoptotic proteins (BCL-2, B-cell lymphoma-extra large [BCL-XL], BCL-W, MCL-1, and BCL-2-related protein A1 [A1/BFL]), play a pivotal role in the regulation of apoptosis by controlling mitochondrial membrane permeability, followed by the release of cytochrome C [30,31]. The BCL-2 family of proteins is differentially expressed in many types of cancer cells to maintain cell survival [32]. Thus, the BCL-2 family is considered a major target for the development of anticancer agents. Consequently, several anticancer drugs, such as venetoclax, obatoclax, and AT-101, which inhibit BCL-2 family proteins, have been developed for the clinical treatment of patients with cancer [33,34].

In this study, we revealed that the expression levels of several apoptotic factors, such as BAX and BCL-2, were commonly upregulated in the three types of latently infected cells compared to those in their parent uninfected cells (Fig 1). In addition, the apoptosis-inducing factor p53 and its downstream genes, *NOXA* and *PUMA*, were upregulated in p53 wild-type ACH2 cells, whereas the upregulation was not observed in p53-defective cells (J1.1 and U1). Several studies have observed the selective killing effect of J_Lat 10.6 cells derived from Jurkat cells (p53 defective) by treatment with a BCL-2 antagonist under reactivation conditions with Ixazomib or anti-CD3/CD28 antibodies [35,36]. p53-defective J1.1 cells exhibited phenotypes that were more resistant to cell death than their parent Jurkat cells under ABT-263 or GX15–070 treatment without a reactivator in our experiments. The apoptotic resistance of p53 defective cells (J1.1) might be due to the overexpression of BCL-2 that exceeds the potency of ABT-263 (Fig 1A and S6 Fig). Therefore, the use of p53 defective cells and reactivation conditions was not considered in our further experiments because they may not be relevant to the physiological infection conditions.

Among the agents that target anti- and pro-apoptotic factors that showed different expression patterns in ACH2 cells from A3.01, the BCL-2 antagonist ABT-263 significantly exerted a selective killing effect on latently infected ACH2 cells. The selective cytotoxic effect of ABT-263 was confirmed by MTT-based cytotoxicity (Fig 3A) and Annexin V-FITC/PI staining assays (Fig 2C). GX15–070 and BP showed slight apoptotic effects on ACH2 cells in the annexin V-FITC/PI staining assay, but no effects were observed in the MTT and sub-G1 assays. These distinctive results might be due to the more delicate detection of the apoptotic cell population using the annexin V-FITC/PI staining assay compared with the sub-G1 and MTT assays. A selective killing effect of GX15–070 and BP was observed in ACH2 cells treated with a combination of ABT-236 (Figs 4 and 5). These data indicate that MCL-1 and XIAP might be assistants in the survival of latently infected cells rather than being essential for cell survival.

SMAC mimetics, such as LCL-161 (SM-406), AT-406 (Debio-1143), and BP, known as therapeutic agents for cancers, induce autophagy-dependent apoptosis of HIV-1-infected macrophages [14] and resting memory CD4^+^ T cells [37]. However, SMAC mimetics alone did not show sufficient selective killing effects in latently infected ACH2 cells (Fig 2). These different effects might be attributed to the lower expression levels of XIAP, FAS, and LC3B in latently HIV-1-infected ACH2 cells compared with the HIV-1-infected macrophages and resting CD4^+^ T cells (Fig 1).

Recently, selective elimination of host cells harboring replication-competent HIV (SECH) based on viral reactivation was reported for the selective killing of latently HIV-1-infected CD4^+^ T cells. SECH approaches have been accomplished by treatment with BCL-2 antagonists combined with autophagy inhibitors [17], BH3 mimetics [38], or integrase inhibitors [39] under viral reactivation. During the reactivation of latently HIV-1-infected T cells, upregulated anti-apoptotic proteins (BCL-XL, MCL-1, and LC3) prevent the apoptosis of the reactivated cells [17]. Therefore, ABT-263 and an autophagy inhibitor synergistically inhibited the survival effects of BCL-XL and LC3 induced in reactivated latently infected T cells, facilitating cell death upon ARV treatment to prevent new infections [17]. However, our study showed that ABT-263 and its combinations selectively induced cell death in latently HIV-1-infected cells without unnecessary processes such as viral reactivation. In addition, ABT-263 and GX-15–070 showed slight or no viral reactivation (S5 Fig). These data revealed that our approach does not require viral reactivation and ARV treatment to inhibit new cell infection.

BCL-2 overexpression is commonly associated with tumor survival and metastatic progression in B-cell lymphoma and breast and pancreatic cancers [40–42]. The BCL-2 antagonist ABT-263 efficiently suppresses B-cell lymphoma and lung cancer cells [43]. The properties of latently HIV-1-infected cells may resemble those of cancer cells expressing high level of BCL-2, allowing ABT-263-sensitive cell death (Fig 2).

ABT-263, AT-101, and GX15–070 are common anti-apoptotic BCL2-family inhibitors, which are known to bind to specific p2 and p4 (p1 and p3 for AT-101) hydrophobic pockets of BCL-XL [44–46], whereas these compounds exhibited the diverse biological activities including different killing effects on cancer cells [44,45] and latently HIV-1-infected cells (Fig 2). Although inhibitory coverage of AT-101 and GX15–070 is broader against the anti-apoptotic BCL-2 family (including A1/BFL-1 and MCL-1) than ABT-263, both inhibitors alone showed a week (or no) selective killing effect on latently infected cells (Fig 2). These data may indicate several probabilities that MCL-1 and A1/BFL-1 are not essential for the survival of latently HIV-1-infeced cells, and/or AT-101 and GX15–070 have weaker affinities for the hydrophobic binding pockets on BCL-XL and BCL-2 than ABT-263, thus causing non-specific effects on some factors regulating the cell-fate of latently HIV-1-infected cells.

Despite the selective killing activity of ABT-263 on the latantly HIV-1-infected cells, the selectivity was not exerted at high doses of ABT-263 (over 10 μM) (S7 Fig), indicating potential cytotoxicity to normal cells. Therefore, the drug ability and optimal dose of ABT-263 should be further determined *in vivo* in animal and clinical studies for therapeutic application.

A high proportion of apoptotic cells was observed among the HIV-1-infected primary CD4^+^ T cells. The apoptotic cell population may be a population of pyroptosis-induced dead cells resulting from massive caspase activation combined with a proinflammatory response caused by abortive infection [29] (Fig 7C).

Latent HIV-1 infection is associated with diverse epigenetic modifications in viral and cellular genomes [47]. In our preliminary ChIP-seq analysis, we revealed that histone activation markers (H3K4me3 and/or H3K9ac) in the promoter regions of BAX and BCL-2 showed more accumulations in ACH2 cells than those in A3.01 cells. This was consistent with the upregulation observed in ACH2 cells. However, the accumulation of activation markers was not observed in the case of XIAP (S8 Fig). Despite the limitations of the obtained data, diverse epigenetic modifications on pro- and anti-apoptotic genes might be associated with the maintenance of survival of latently HIV-1-infected cells; the detailed mechanism remains to be unveiled. A summarized strategy for the selective killing of latently HIV-1-infected cells, as suggested in our study, is illustrated schematically (S9 Fig).

## Conclusion

Our data demonstrate that the BCL-2 antagonist ABT-263 and its combinations could selectively kill latently HIV-1-infected cells without viral reactivation. These findings might be helpful in developing a novel therapeutic approach to eliminate latently infected HIV-1 cells.

## Supporting information

S1 Fig Autophagic phenotypes of latently HIV-1 infected cells. Latently HIV-1- infected cells (ACH2, J1.1, and U1) and their parent cells (A3.01, Jurkat, and U937) were cultured for 24 h in 6-well plates. The cells were fixed with 4% paraformaldehyde. Subsequently, the cells were incubated with an anti-LC3B antibody followed by an anti-mouse IgG conjugated with Alexa-488. The accumulated LC3B in the cells was visualized using fluorescence microscopy (Olympus IX-83, Shinjuku, Japan) at x 400 magnification. The increased LC3B signal in A3.01 cells following chloroquine (CQ) treatment indicates a mature autophagosome. DAPI was used to stain the cell nuclei.
(PDF)

S2 Fig mRNA expression of apoptosis-regulating factors in latently HIV-1-infected cells. Relative mRNA levels of apoptosis-regulating factors in A3.01 and latently HIV-1-infected ACH2 cells were determined using RT-qPCR. The expression of each sample was normalized to that of GAPDH. The data are expressed as mean ± SD (n = 3). *p < 0.05, **p < 0.01, compared with A3.01 cells.
(PDF)

S3 Fig Selective killing effects of ABT-263 and its combination on latently HIV-1-infected cells. The cells were treated with ABT-263 (1 μM) or its combinations with SMBA1 (1 μM), BTSA1 (1 μM), AT-101 (1 μM), GX15–070 (1 μM), AT-406 (1 μM), BP (1 μM), and LCL-161 (1 μM), respectively. At 24 h after treatment, the sub-G1 cell population was determined using flow cytometry after PI staining. Data are expressed as mean ± SD (n = 3). **p < 0.01, compared with A3.01 cells.
(PDF)

S4 Fig Selective apoptotic effects of ABT-263 and etoposide on latently HIV-1 infected ACH2 cells. The cells were treated with ABT-263 (1 μM), etoposide (ES) (1 μM), or a combination of both drugs for 24 h. The early and late phases of apoptotic cells were then analyzed by flow cytometry using annexin V-FITC/PI staining.
(PDF)

S5 Fig Viral reactivation of apoptosis inducing agents. (A) ACH2 cells were treated with apoptosis inducing agents (each 5 μM and 1 μg/mL of PMA) for 48 h. The intracellular level of p24 was detected by Western blotting analysis. (B) The secreted p24 level was determined by the ALPHA ELISA assay under the same experimental conditions. The data are expressed as mean ± SD (n = 3). **p < 0.01, compared with DMSO control.
(PDF)

S6 Fig Apoptosis inducing effects of ABT-263 and GX15–070 on Jurkat and latently HIV-1-infected J1.1 cells. (A) Jurkat and J1.1 cells were treated with ABT-260, GX15–070, or ABT-263 combined with GX15–070 (all concentrations, 1 μM). At 24 h after treatment, expression of proteins was analyzed by Western blotting using the indicated antibodies and β-actin as a loading control. (B) The cells were treated with ABT-263 (1 μM) ES (1 μM) for 24 h. The early and late phases of apoptotic cells were then analyzed by flow cytometry using annexin V-FITC/PI staining.
(PDF)

S7 Fig Cell viability of latently HIV-1-infected ACH2 cells and their non-infected parent A3.01 cells upon ABT-263 treatment. A3.01 and ACH2 cells were treated with ABT-263 at the indicated concentrations for 24 h, and cell viability was determined using an MTT assay. Data are represented as the relative mean value ± SD (n = 3) compared with the DMSO control. *p < 0.05, **p < 0.01, compared with DMSO control.
(PDF)

S8 Fig Histone chip-seq analysis of apoptosis-inducing factors. Histone activation markers (H3K4me3 and H3K9ac) of apoptosis-inducing factors (BAX, BCL-2, and XIAP) were analyzed in latently infected ACH2 cells and their non-infected parent A3.01 cells. The upper panel shows the aligned histone activation markers for each gene on the chromosome, and the lower panel presents the relative intensity of these markers in the promoter region of each gene.
(PDF)

S9 Fig Schematic strategy for selective killing of latently HIV-1-infected cells. Latently HIV-1-infected cells express higher levels of apoptosis-regulators (BAX, BCL-2, and XIAP) than those expressed by non-infected parent cells. The expression pattern of these factors may contribute to maintaining the survival of latently HIV-1-infected cells in spite of sustained genotoxic proviral infection. Treatment with ABT-263 facilitates the activity of pro-apoptotic proteins (including BAX and BAK) by inhibiting BCL-2, followed by the release of cytochrome C from mitochondria in latently HIV-1-infected cells. Furthermore, treatment with GX-15–070 (which inhibits anti-apoptotic MCL-1), BTSA-1 (which promotes BAX oligomerization), and BP (which inhibits IAPs) strengthens the ABT-263-induced apoptotic pathway, thereby increasing the selective killing effect of ABT-263 on latently HIV-1-infected cells.
(PDF)

S1 Table Apoptosis-inducing agents and antibodies. (DOCX)

S1 Raw Images (PDF)
