# Supplementary figures and images for: ABT-263, a BCL-2 inhibitor, selectively eliminates latently HIV-1-infected cells without viral reactivation
Source: PLoS One. 2025 May 20;20(5):e0322962. doi: 10.1371/journal.pone.0322962 (PMC12091775; doi:10.1371/journal.pone.0322962)

S1 Figure

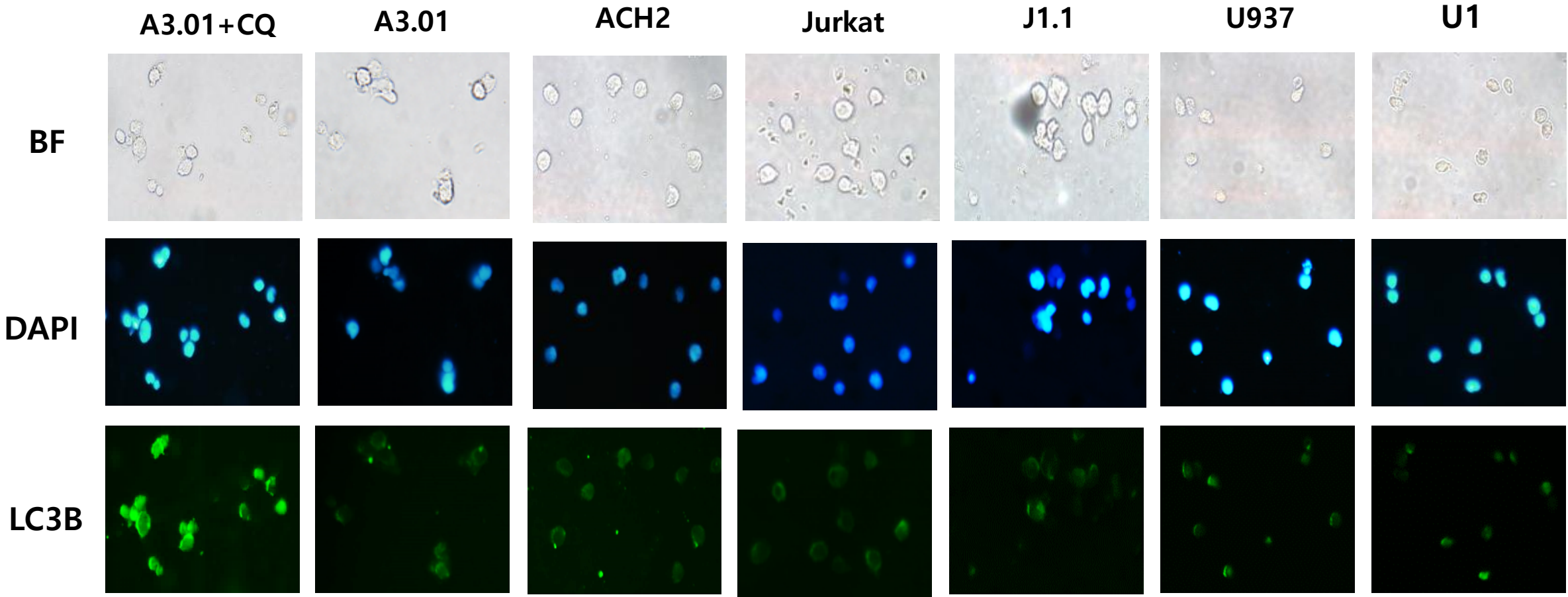

Supplement: S1 Fig — Latently HIV-1- infected cells (ACH2, J1.1, and U1) and their parent cells (A3.01, Jurkat, and U937) were cultured for 24 h in 6-well plates. The cells were fixed with 4% paraformaldehyde. Subsequently, the cells were incubated with an anti-LC3B antibody followed by an anti-mouse IgG conjugated with Alexa-488. The accumulated LC3B in the cells was visualized using fluorescence microscopy (Olympus IX-83, Shinjuku, Japan) at x 400 magnification. The increased LC3B signal in A3.01 cells following chloroquine (CQ) treatment indicates a mature autophagosome. DAPI was used to stain the cell nuclei. (PDF) [file pone.0322962.s001.pdf]

S2 Figure

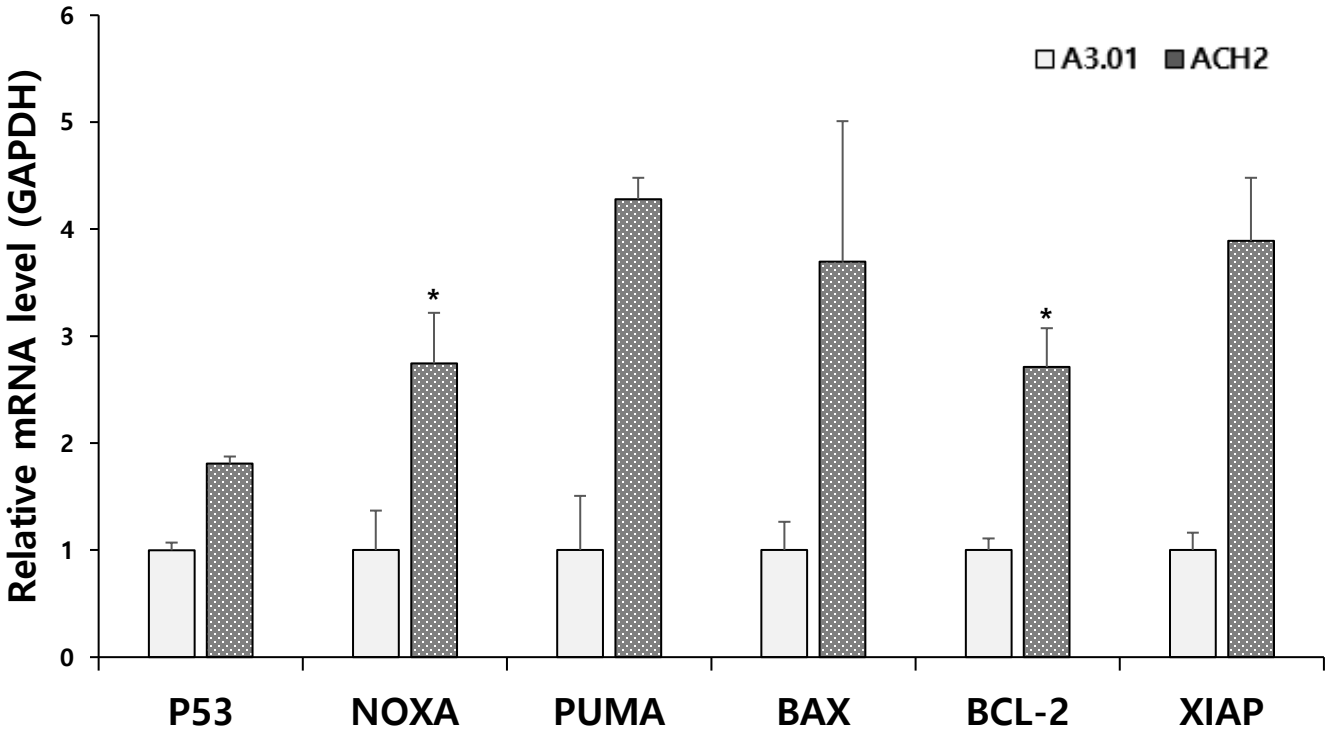

Supplement: S2 Fig — Relative mRNA levels of apoptosis-regulating factors in A3.01 and latently HIV-1-infected ACH2 cells were determined using RT-qPCR. The expression of each sample was normalized to that of GAPDH. The data are expressed as mean ± SD (n = 3). *p < 0.05, **p < 0.01, compared with A3.01 cells. (PDF) [file pone.0322962.s002.pdf]

S3 Figure

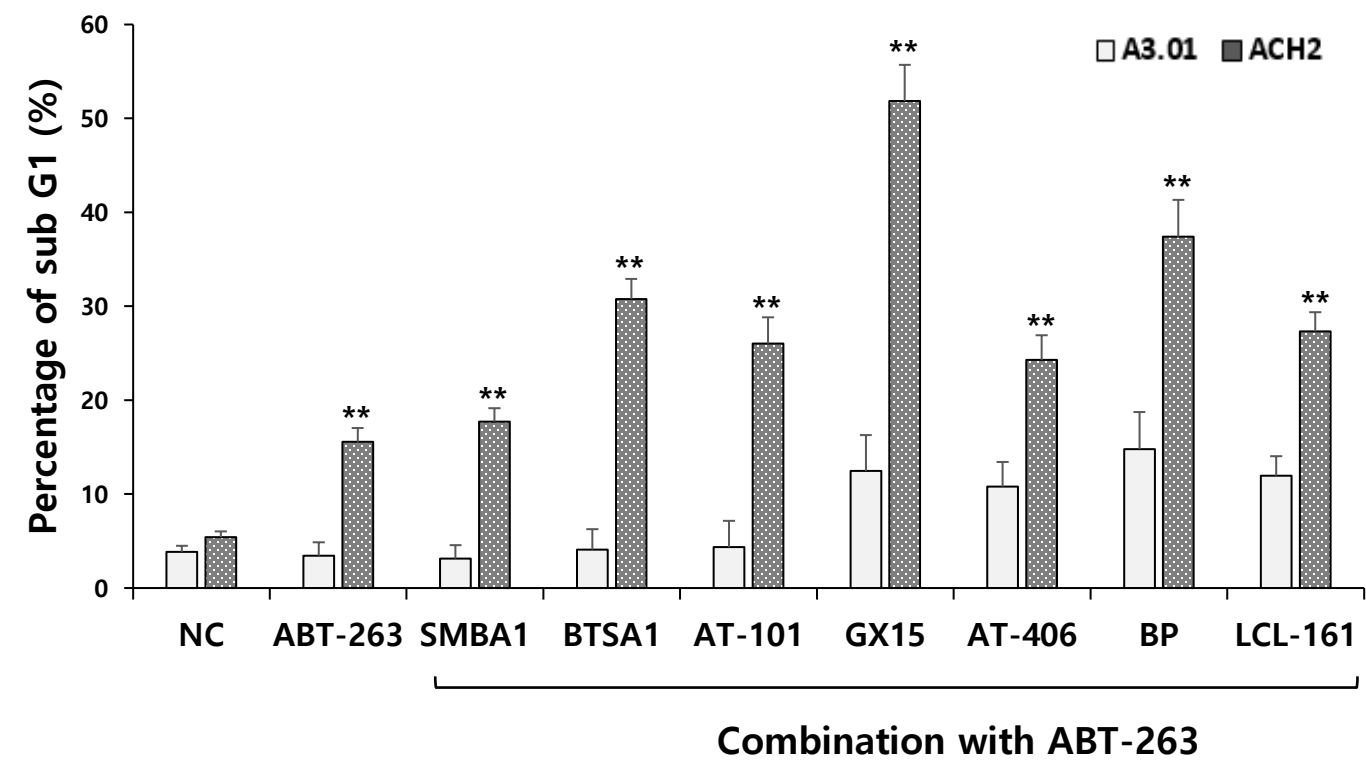

Supplement: S3 Fig — The cells were treated with ABT-263 (1 μM) or its combinations with SMBA1 (1 μM), BTSA1 (1 μM), AT-101 (1 μM), GX15–070 (1 μM), AT-406 (1 μM), BP (1 μM), and LCL-161 (1 μM), respectively. At 24 h after treatment, the sub-G1 cell population was determined using flow cytometry after PI staining. Data are expressed as mean ± SD (n = 3). **p < 0.01, compared with A3.01 cells. (PDF) [file pone.0322962.s003.pdf]

S4 Figure

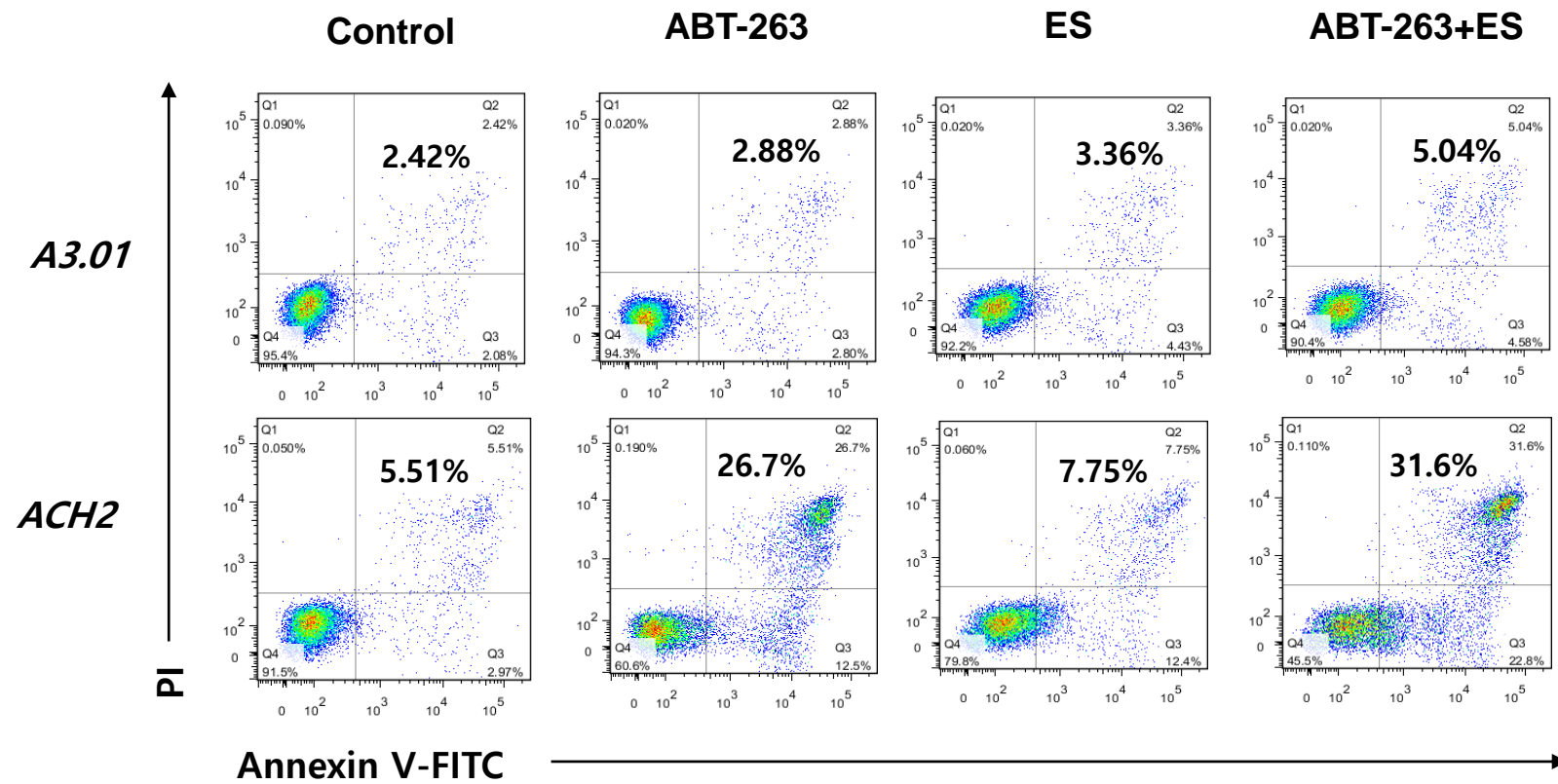

Supplement: S4 Fig — The cells were treated with ABT-263 (1 μM), etoposide (ES) (1 μM), or a combination of both drugs for 24 h. The early and late phases of apoptotic cells were then analyzed by flow cytometry using annexin V-FITC/PI staining. (PDF) [file pone.0322962.s004.pdf]

S5 Figure

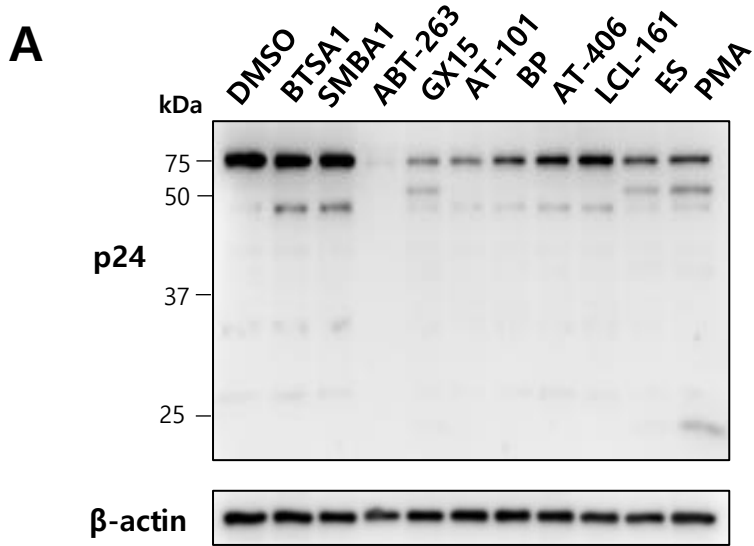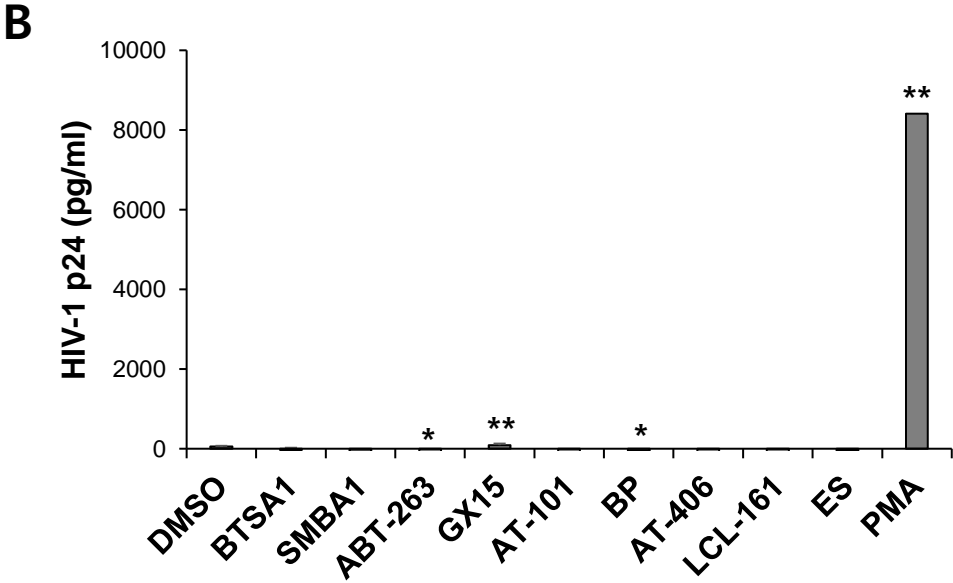

Supplement: S5 Fig — (A) ACH2 cells were treated with apoptosis inducing agents (each 5 μM and 1 μg/mL of PMA) for 48 h. The intracellular level of p24 was detected by Western blotting analysis. (B) The secreted p24 level was determined by the ALPHA ELISA assay under the same experimental conditions. The data are expressed as mean ± SD (n = 3). **p < 0.01, compared with DMSO control. (PDF) [file pone.0322962.s005.pdf]

S6 Figure

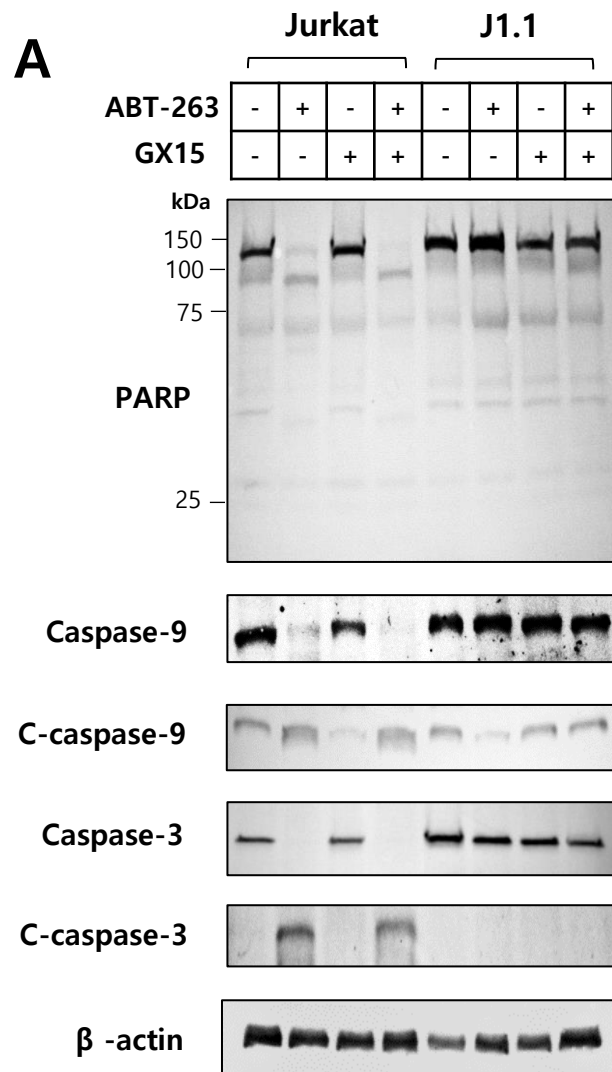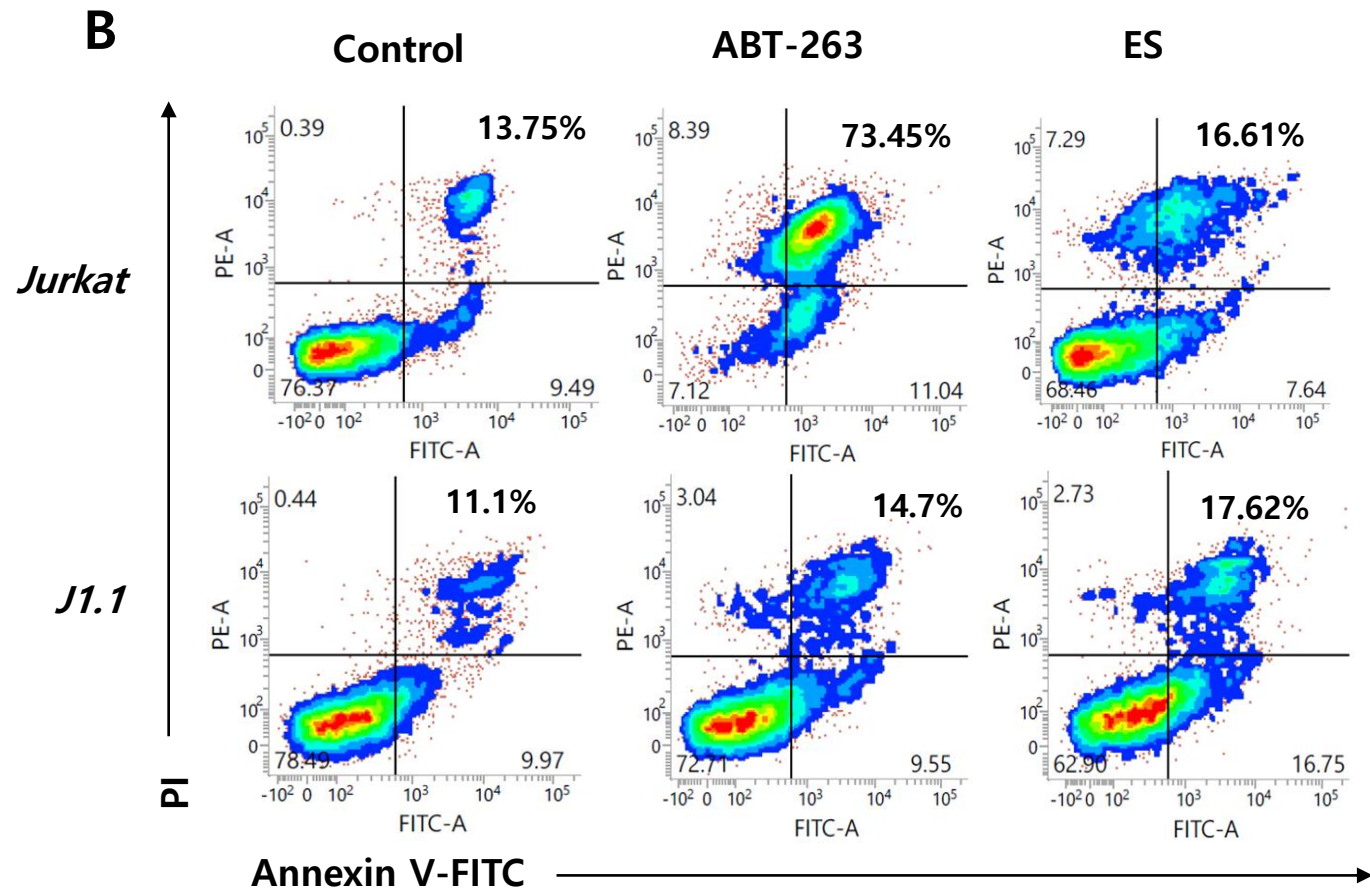

Supplement: S6 Fig — (A) Jurkat and J1.1 cells were treated with ABT-260, GX15–070, or ABT-263 combined with GX15–070 (all concentrations, 1 μM). At 24 h after treatment, expression of proteins was analyzed by Western blotting using the indicated antibodies and β-actin as a loading control. (B) The cells were treated with ABT-263 (1 μM) ES (1 μM) for 24 h. The early and late phases of apoptotic cells were then analyzed by flow cytometry using annexin V-FITC/PI staining. (PDF) [file pone.0322962.s006.pdf]

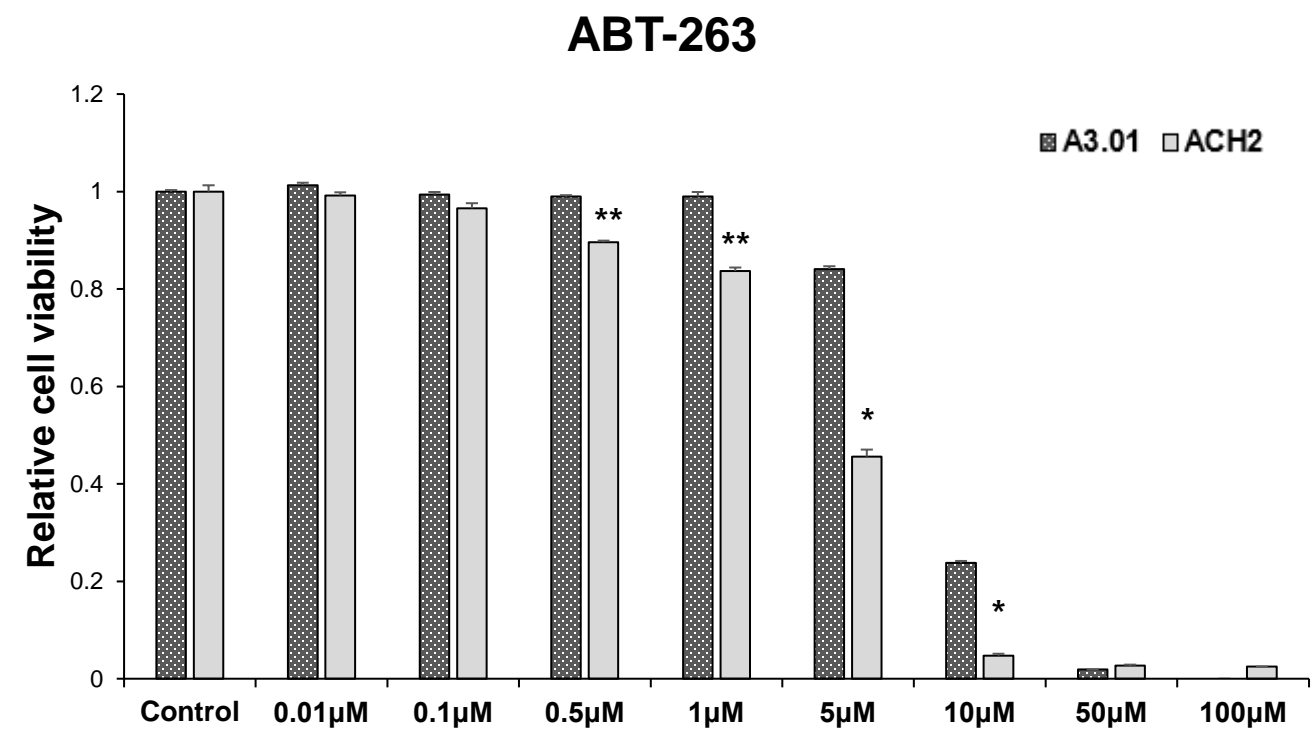

Supplement: S7 Fig — A3.01 and ACH2 cells were treated with ABT-263 at the indicated concentrations for 24 h, and cell viability was determined using an MTT assay. Data are represented as the relative mean value ± SD (n = 3) compared with the DMSO control. *p < 0.05, **p < 0.01, compared with DMSO control. (PDF) [file pone.0322962.s007.pdf]

S8 Figure

**BAX**

chr19: 48,953,248 - 48,974,019 (20,772bp)

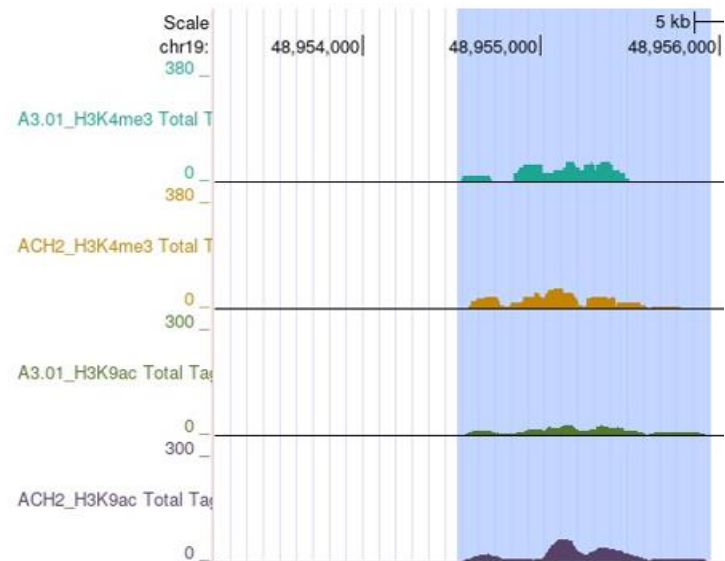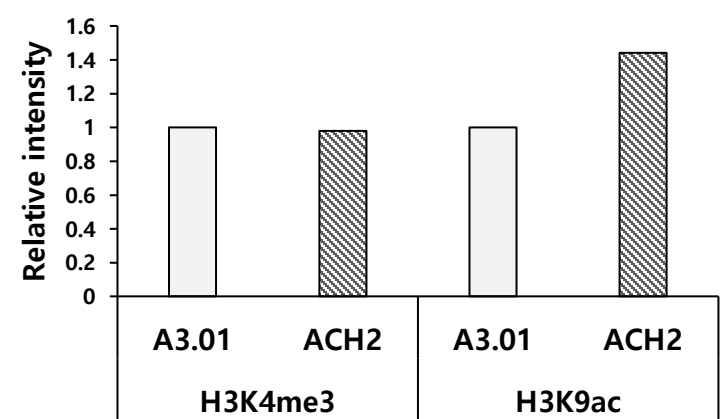

**BCL-2**

chr18: 62,926,922 - 63,516,193 (589,272bp)

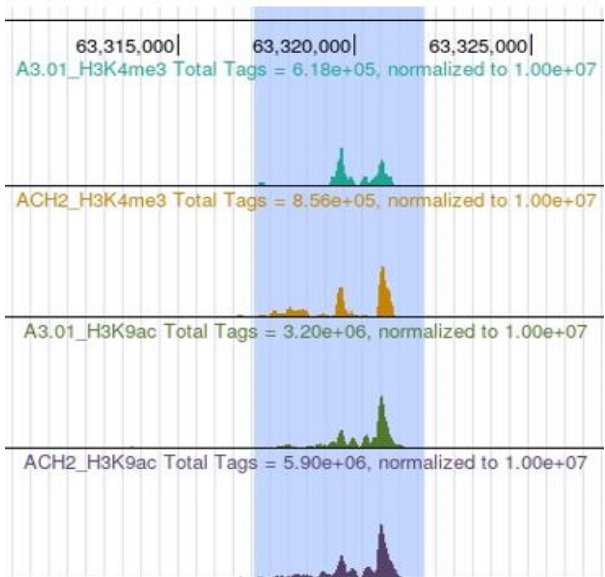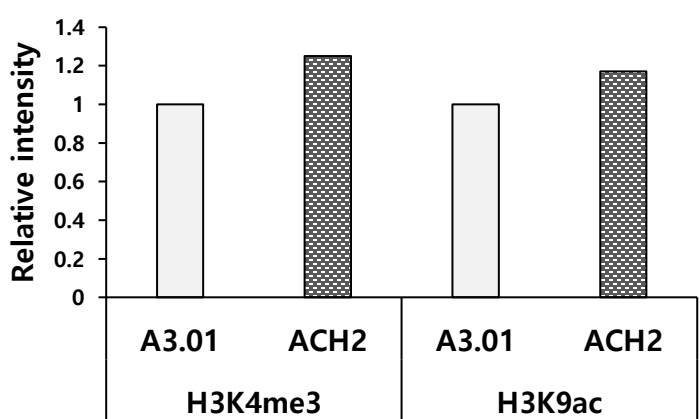

**XIAP**

chrX: 123,858,559 - 123,876,528 (17,973bp)

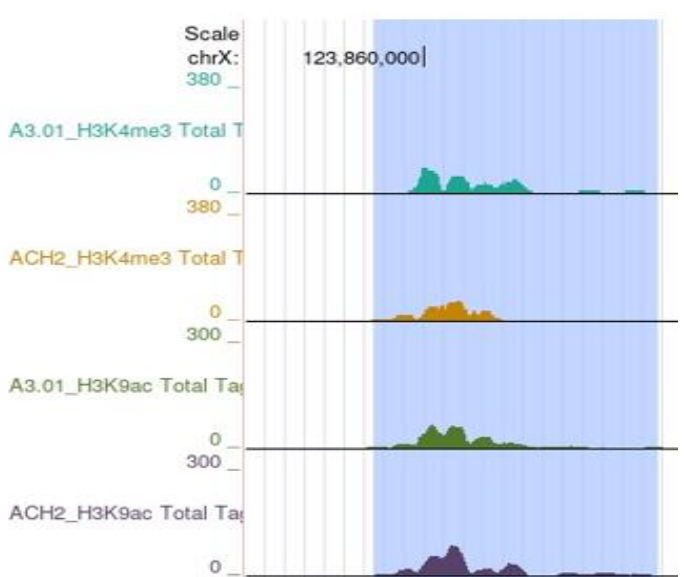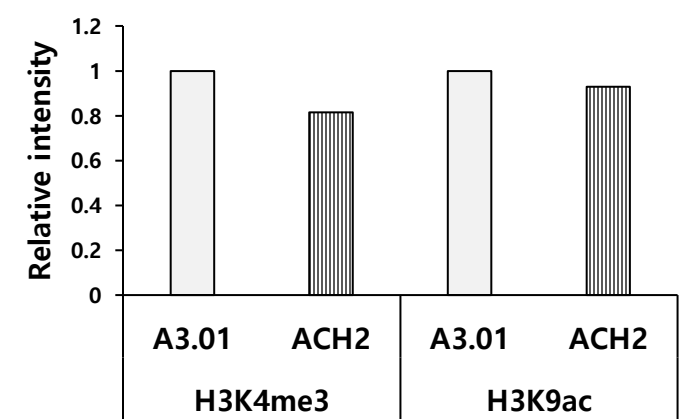

Supplement: S8 Fig — Histone activation markers (H3K4me3 and H3K9ac) of apoptosis-inducing factors (BAX, BCL-2, and XIAP) were analyzed in latently infected ACH2 cells and their non-infected parent A3.01 cells. The upper panel shows the aligned histone activation markers for each gene on the chromosome, and the lower panel presents the relative intensity of these markers in the promoter region of each gene. (PDF) [file pone.0322962.s008.pdf]

S9 Figure

Latently HIV-1 infected cell

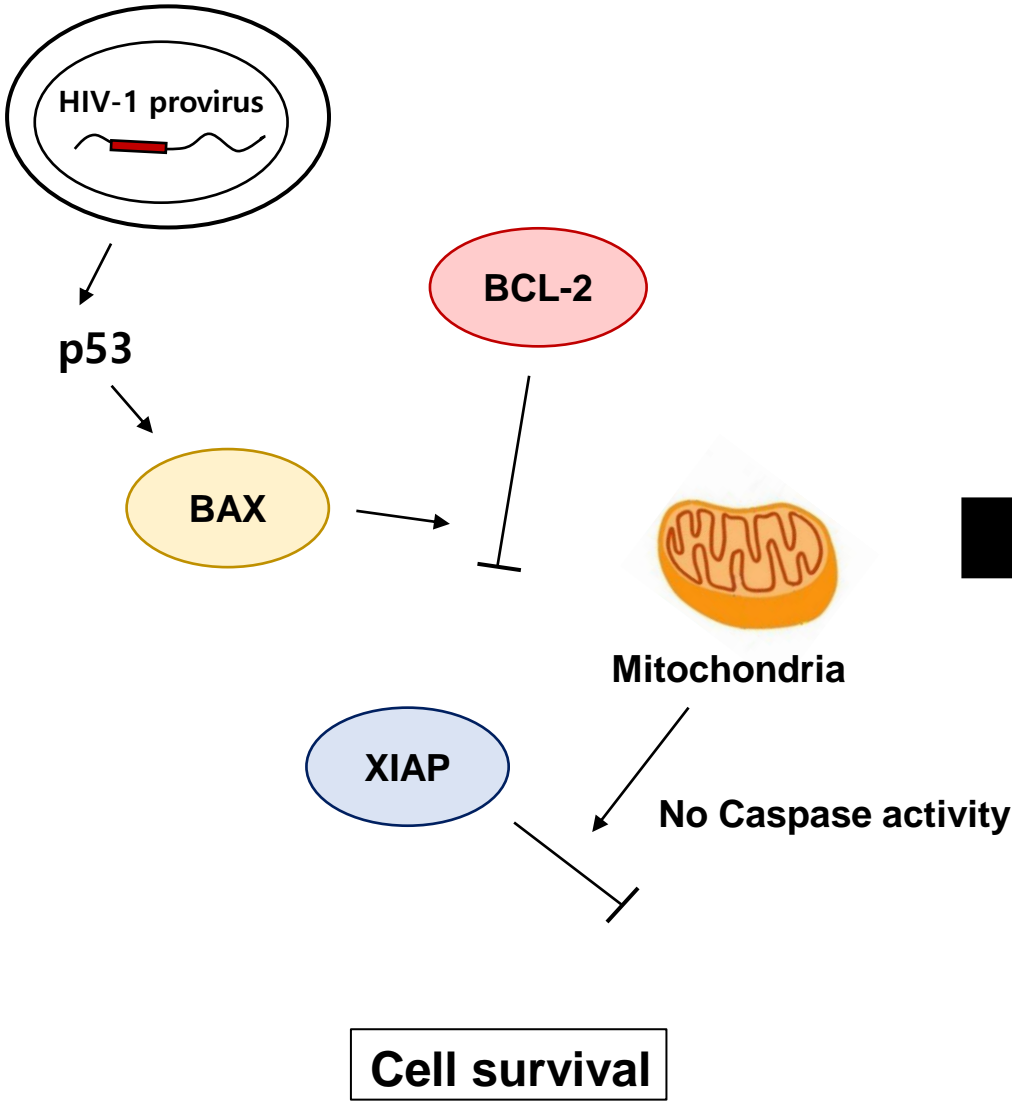

Latently HIV-1 infected cell

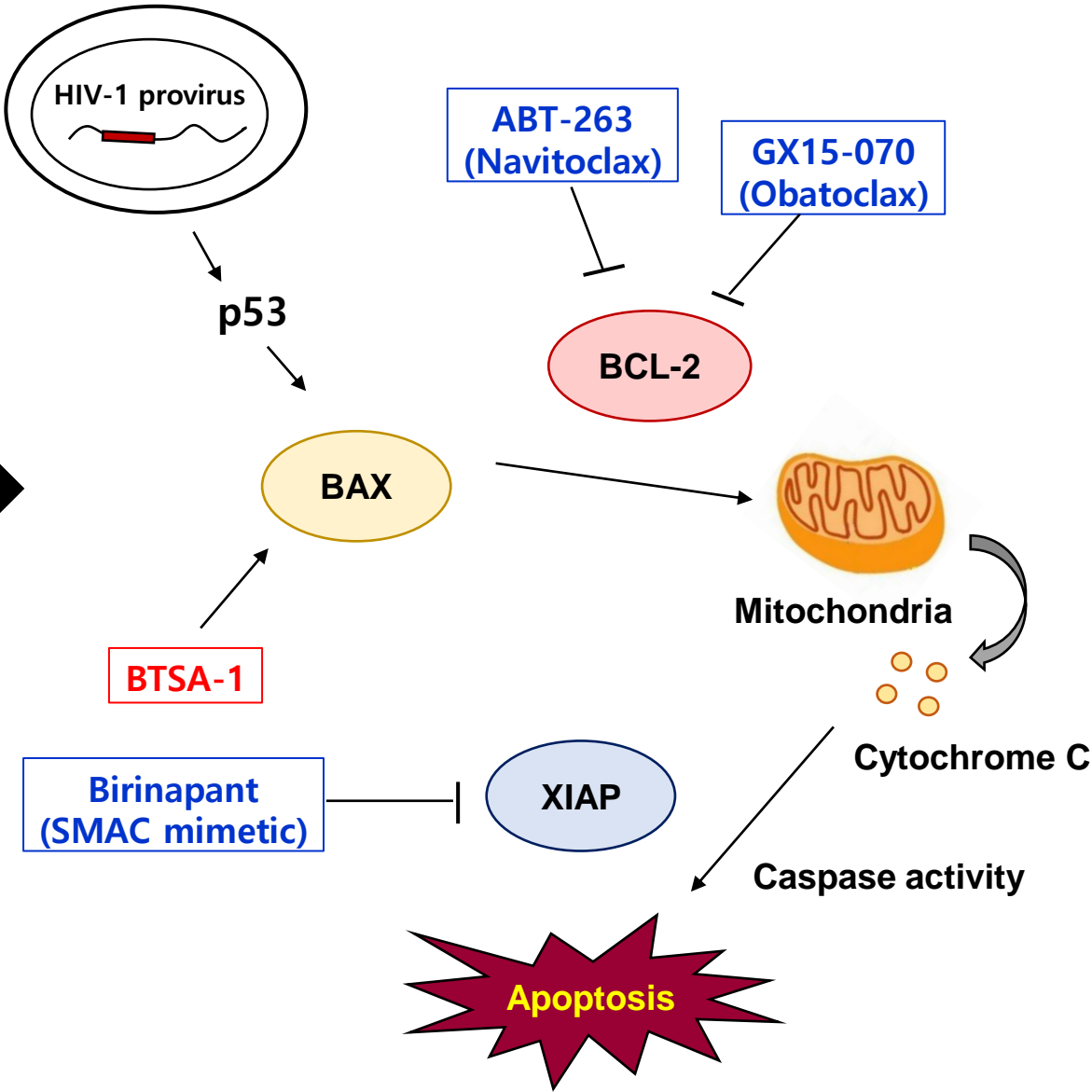

Supplement: S9 Fig — Latently HIV-1-infected cells express higher levels of apoptosis-regulators (BAX, BCL-2, and XIAP) than those expressed by non-infected parent cells. The expression pattern of these factors may contribute to maintaining the survival of latently HIV-1-infected cells in spite of sustained genotoxic proviral infection. Treatment with ABT-263 facilitates the activity of pro-apoptotic proteins (including BAX and BAK) by inhibiting BCL-2, followed by the release of cytochrome C from mitochondria in latently HIV-1-infected cells. Furthermore, treatment with GX-15–070 (which inhibits anti-apoptotic MCL-1), BTSA-1 (which promotes BAX oligomerization), and BP (which inhibits IAPs) strengthens the ABT-263-induced apoptotic pathway, thereby increasing the selective killing effect of ABT-263 on latently HIV-1-infected cells. (PDF) [file pone.0322962.s009.pdf]
