# Supplementary material for: ABT-263, a BCL-2 inhibitor, selectively eliminates latently HIV-1-infected cells without viral reactivation
Source: PLoS One. 2025 May 20;20(5):e0322962. doi: 10.1371/journal.pone.0322962 (PMC12091775; doi:10.1371/journal.pone.0322962)
Supplement: S1 Table — (DOCX) [file pone.0322962.s010.docx]

**S1 Table. Information of apoptosis inducing agents and antibodies**

| **Antibodies/ Chemicals** | **Company** | **Catalog no** |
| --- | --- | --- |
| α-TP53 | Santacruz biotechnology | sc-126 |
| α-BAX | Santacruz biotechnology | sc-7480 |
| α-BID | Santacruz biotechnology | sc-373939 |
| α-BAD | Santacruz biotechnology | 9239 |
| α-BIM | Santacruz biotechnology | 2933 |
| α-NOXA | Santacruz biotechnology | sc-59169 |
| α-PUMA | Santacruz biotechnology | sc-374223 |
| α-BCL-2 | Santacruz biotechnology | sc-7382 |
| α-BCL-XL | Santacruz biotechnology | sc-8392 |
| α-MCL-1 | Santacruz biotechnology | sc-12756 |
| α-XIAP | Santacruz biotechnology | sc-55550 |
| α-SMAC | Santacruz biotechnology | sc-393118 |
| α-Caspase-9 | Cell signaling technology | 6504S |
| α-C-caspase-9 | Cell signaling technology | 20750S |
| α-Caspase-3 | Cell signaling technology | 9662S |
| α-C-caspase-3 | Cell signaling technology | 96661S |
| α-Caspase-7 | Cell signaling technology | 12827T |
| α-Caspase-8 | Santacruz biotechnology | sc-56070 |
| α-PARP | Cell signaling technology | 9542S |
| α-FAS | Santacruz biotechnology | sc-8009 |
| α-FASL | Santacruz biotechnology | sc-19681 |
| α-MLKL | Cell signaling technology | 14993 |
| α-RIP | Santacruz biotechnology | sc-133102 |
| α-LC3B | Santacruz biotechnology | sc-271625 |
| α-Cytochrome C | Cell signaling technology | 11940S |
| α-Beta actin | Santacruz biotechnology | sc-47778 |
| α-COX-4 | Santacruz biotechnology | sc-376731 |
| BTSA1 | Selleckchem | S8650 |
| SMBA1 | Sigma | SNK1464 |
| ABT-263 (Navitoclax) | Selleckchem | S1001 |
| AT-101 | Tocris bioscience | 3367 |
| GX15-070 (Obatoclax) | Sigma | SML3118 |
| AT-406 | Selleckchem | s2754 |
| Birinapant | Selleckchem | s7015 |
| LCL-161 | Selleckchem | s7009 |
| Etoposide | Cell signaling technology | 2200S |
