# Supplementary material for: ABT-263, a BCL-2 inhibitor, selectively eliminates latently HIV-1-infected cells without viral reactivation
Source: PLoS One. 2025 May 20;20(5):e0322962. doi: 10.1371/journal.pone.0322962 (PMC12091775; doi:10.1371/journal.pone.0322962)

Fig 1A_p53

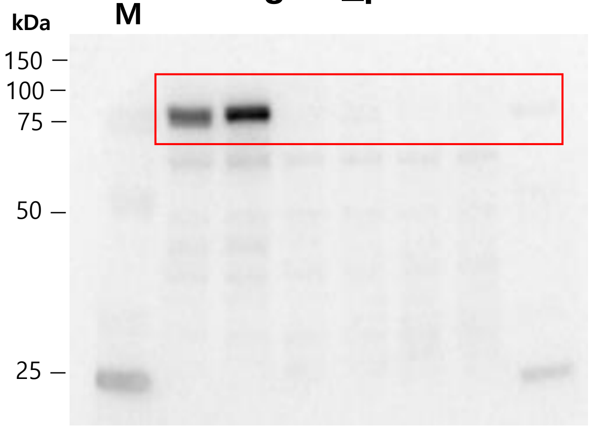

Fig 1A_BAX

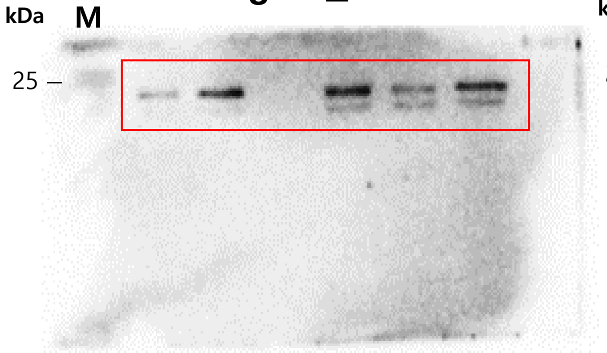

Fig 1A_BID

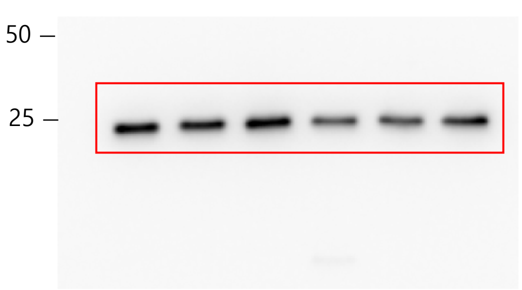

Fig 1A_BAD

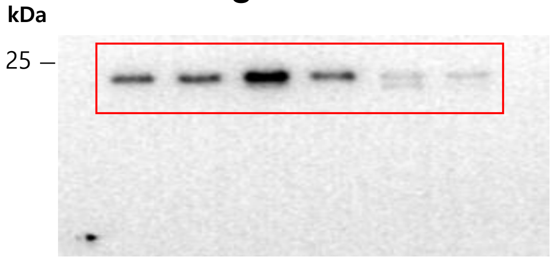

Fig 1A_BIM

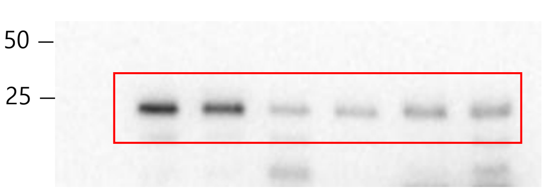

Fig 1A_NOXA

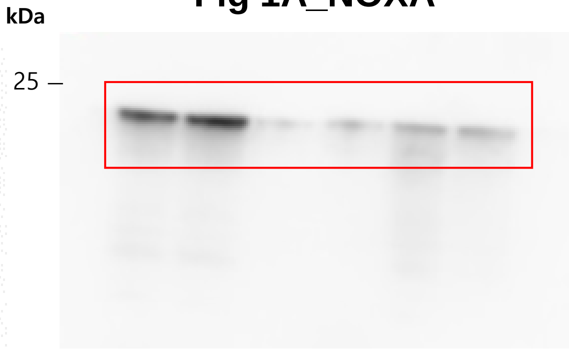

Fig 1A_PUMA

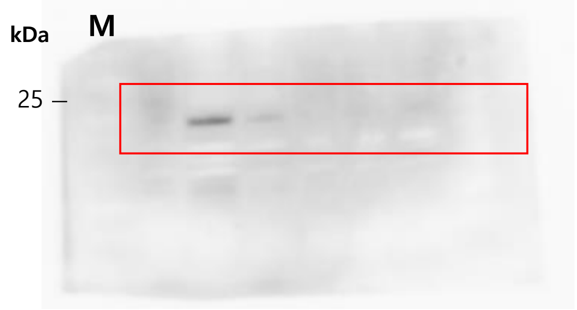

Fig 1A_SMAC

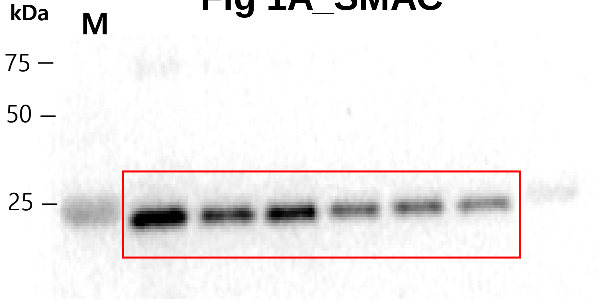

Fig 1A_BCL-2

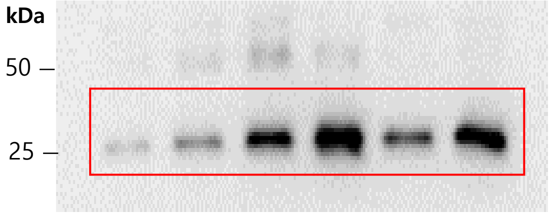

Fig 1A_MCL-1

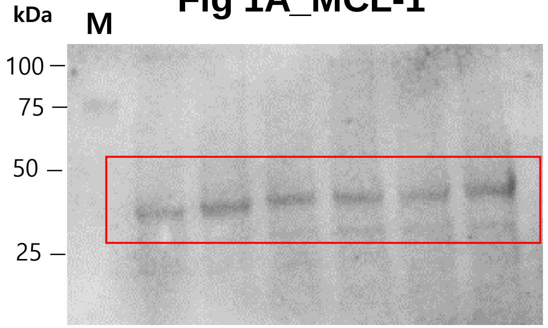

Fig 1A_XIAP

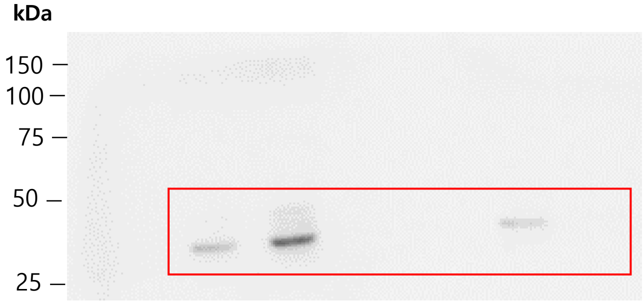

Fig 1A_Caspase-9

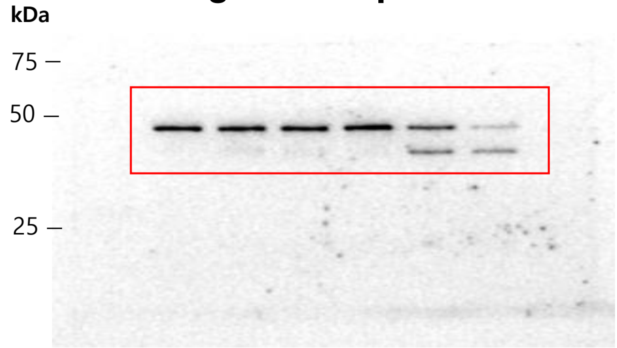

Fig 1A_Caspase-3

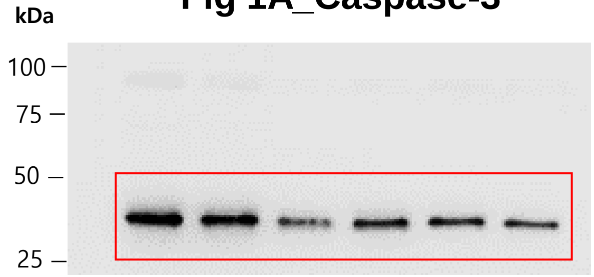

Fig 1A_Caspase-7

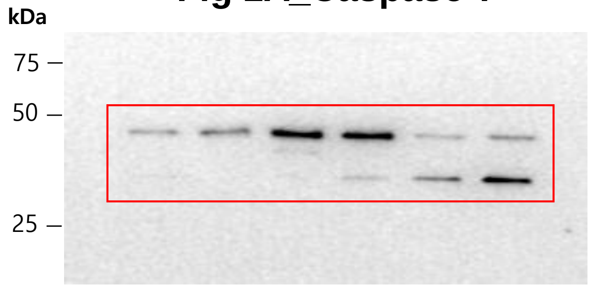

Fig 1A_PARP

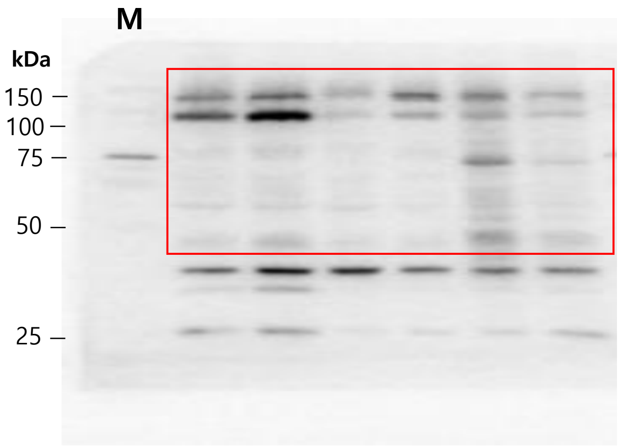

Fig 1A_β-actin

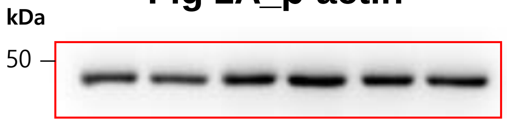

Fig 1B_FAS

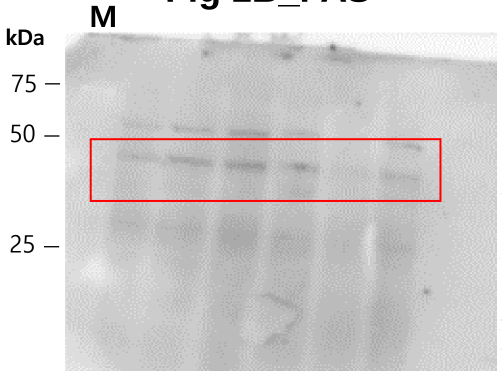

Fig 1B_FASL

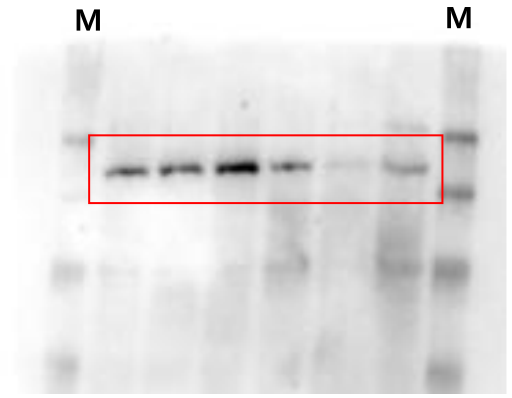

Fig 1B_MLKL

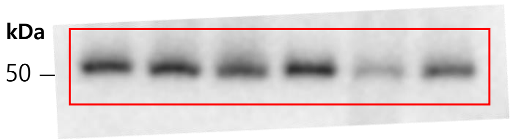

Fig 1B_RIP

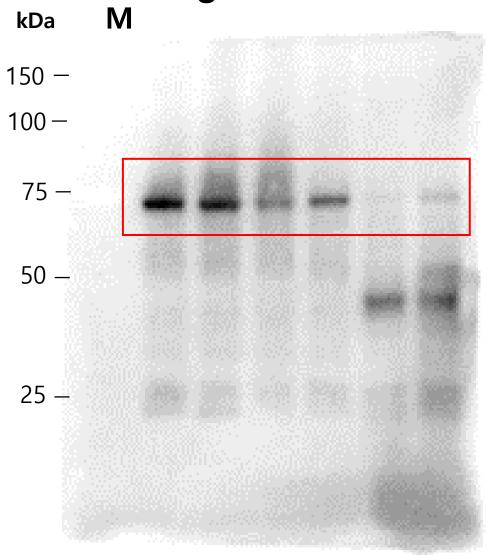

Fig 1B_Caspase-8

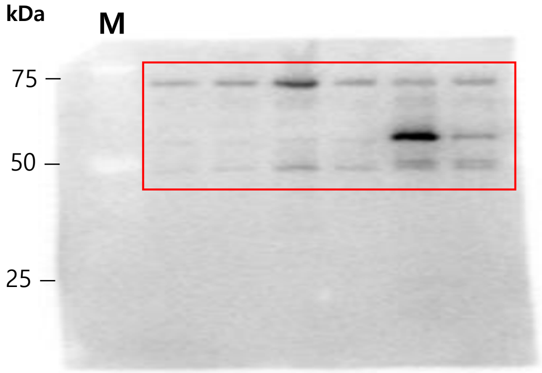

Fig 1B_β-actin

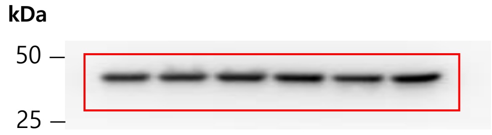

Fig 1C_LC3B

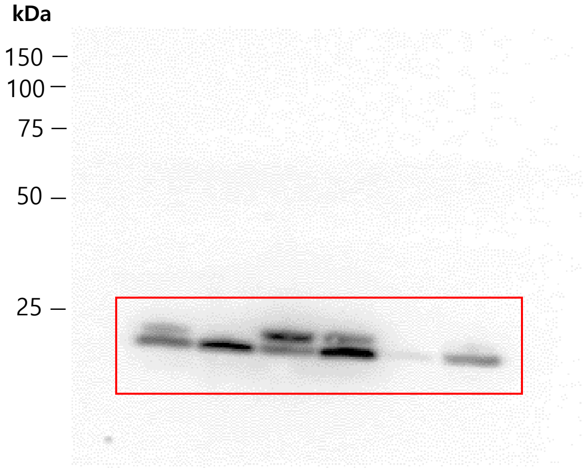

Fig 1C_β-actin

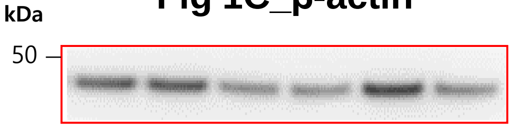

Fig 2E_PARP

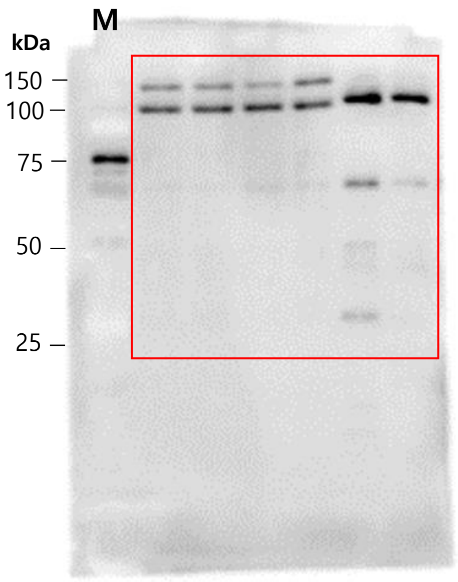

Fig 2E_C-caspase-9

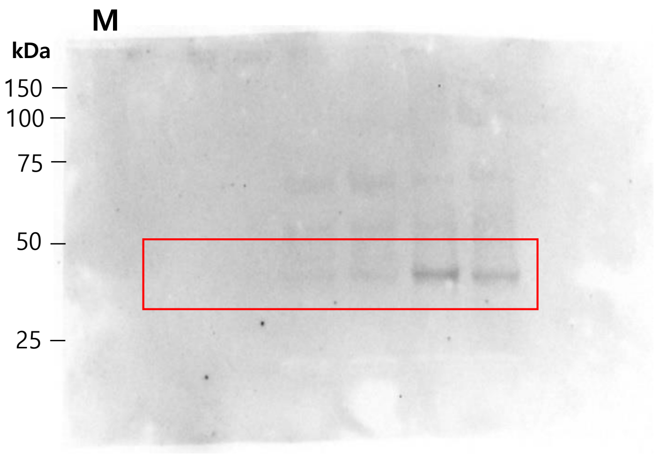

Fig 2E_C-caspase-3

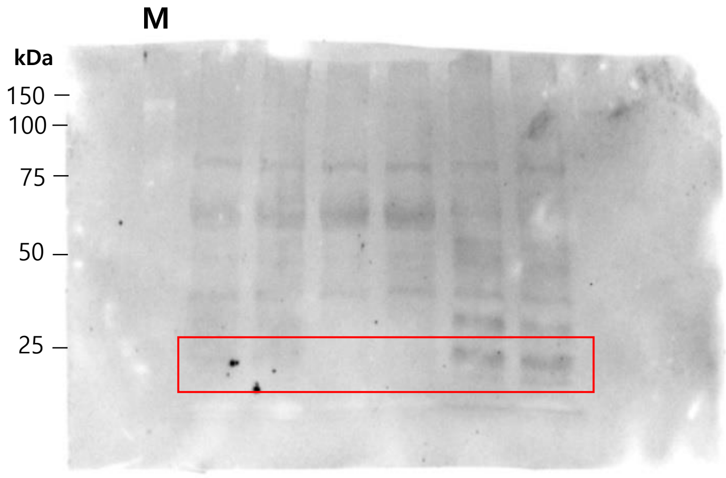

Fig 2E_Caspase-9

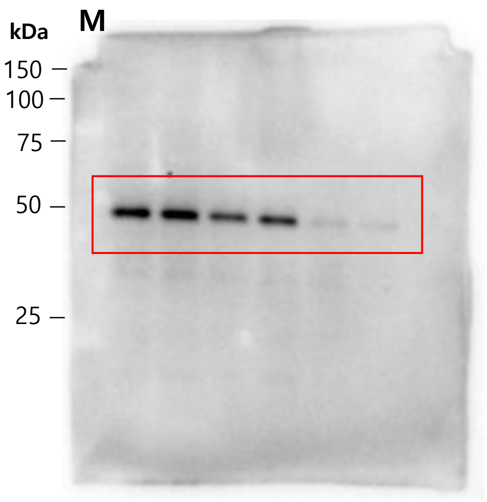

Fig 2E_Caspase-3

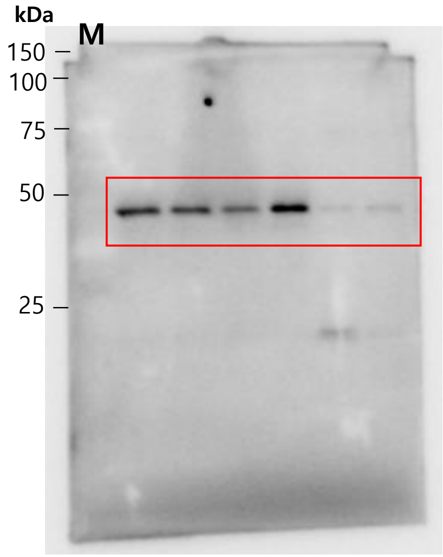

Fig 2E_β-actin

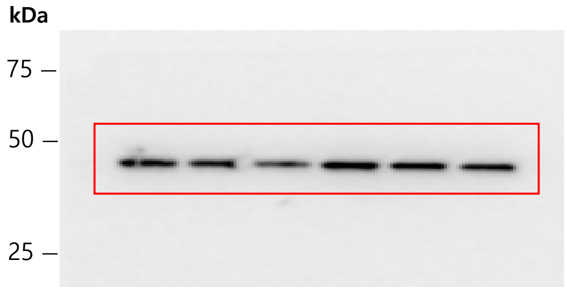

Fig 3C_PARP

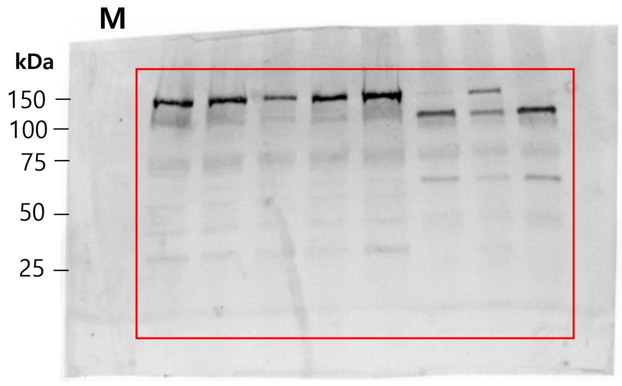

Fig 3C_C-caspase-9

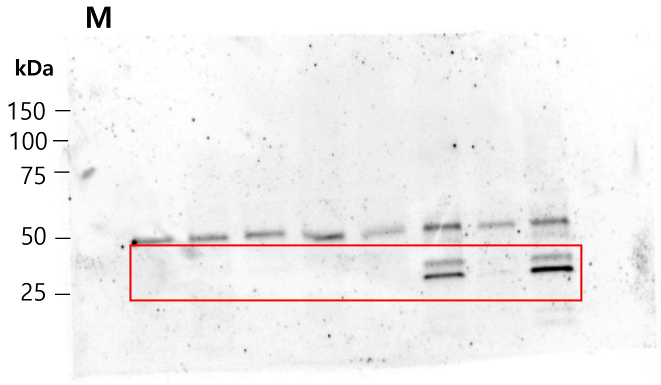

Fig 3C_C-caspase-3

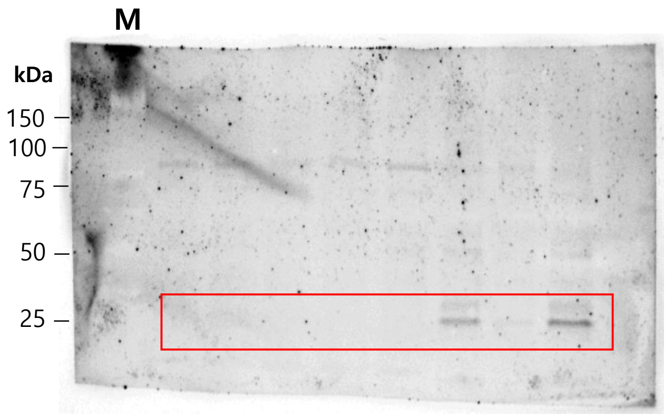

Fig 3C_Caspase-9

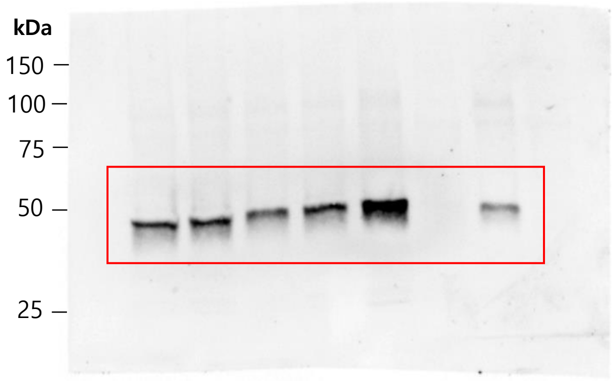

Fig 3C_Caspase-3

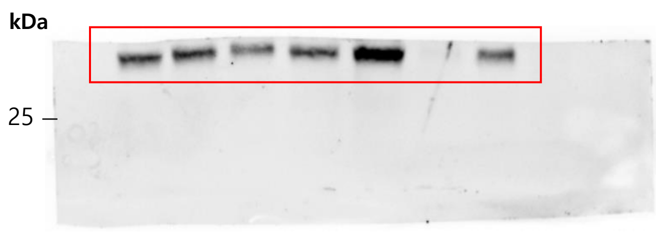

Fig 3C_β-actin

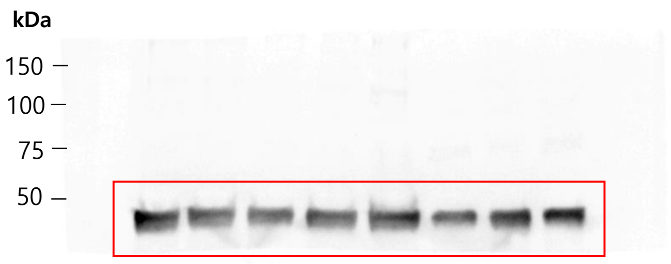

ABT-263 + BTSA1

Fig 4C_Cyto C (Cyto)

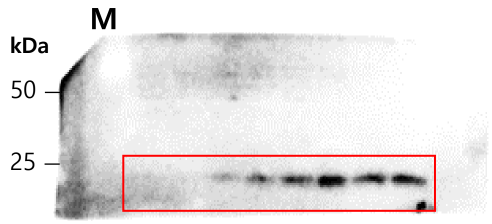

Fig 4C_β-actin (Cyto)

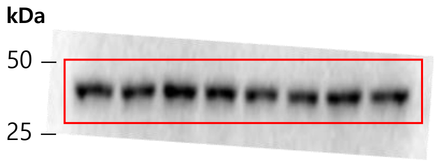

Fig 4C_Cyto C (Mito)

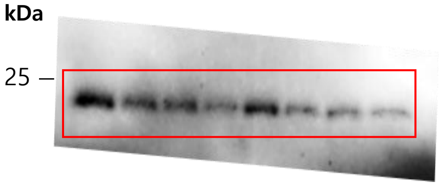

Fig 4C_COX IV (Mito)

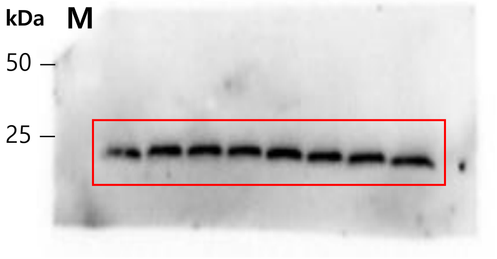

ABT-263 + GX15

Fig 4C_Cyto C (Cyto)

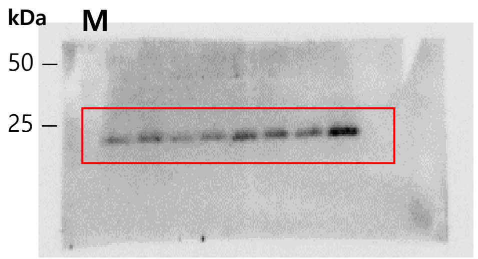

Fig 4C_β-actin (Cyto)

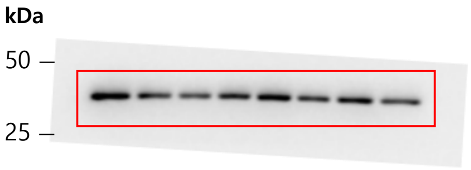

Fig 4C_Cyto C (Mito)

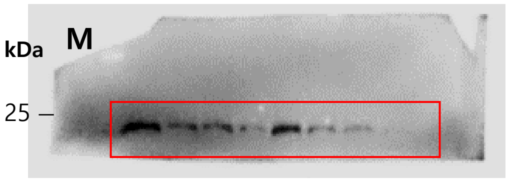

Fig 4C_COX IV (Mito)

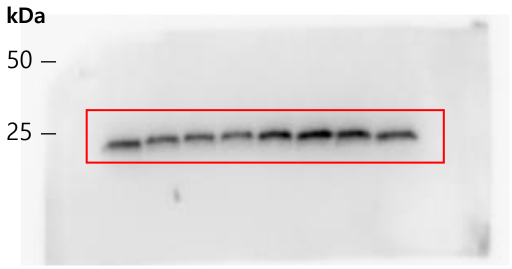

Fig 5A_p24

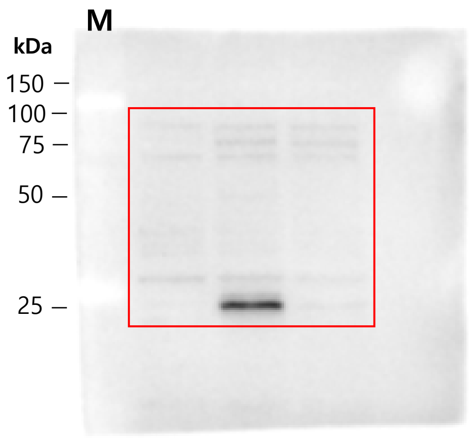

Fig 5A_BAX

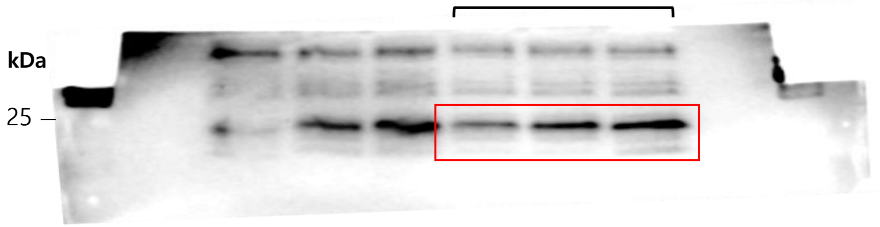

Fig 5A_BCL-2

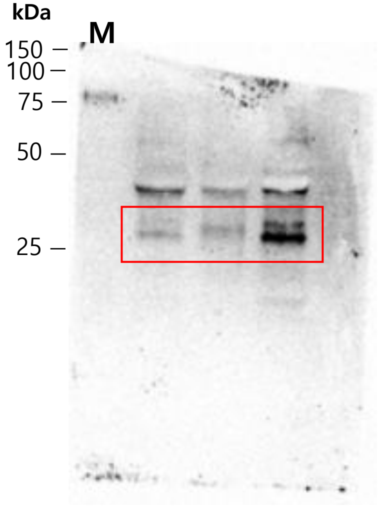

Fig 5A_XIAP

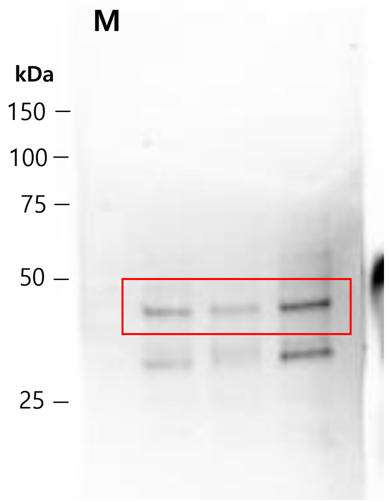

Fig 5A_MCL-1

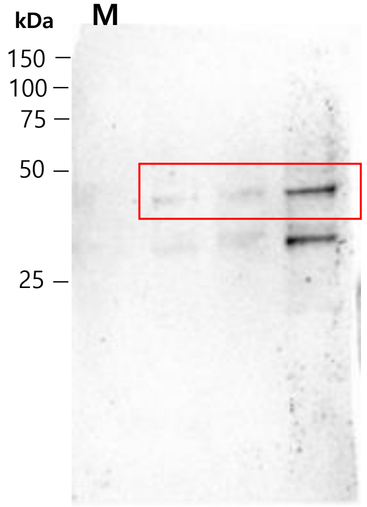

Fig 5A_SMAC

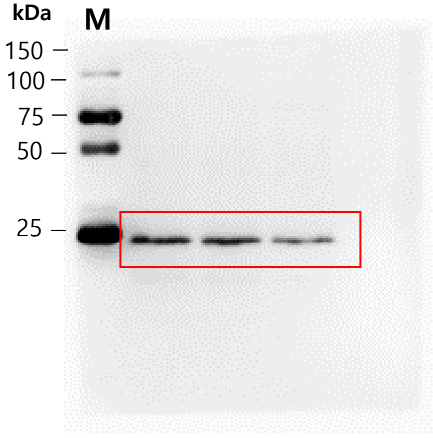

Fig 5A_β-actin

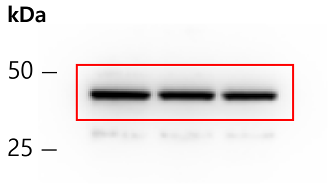

Fig S5A_p24

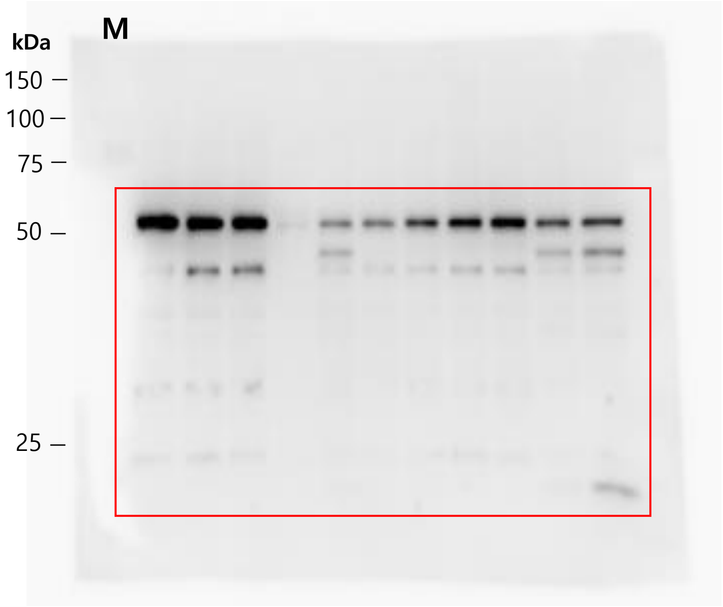

Fig S5B_β-actin

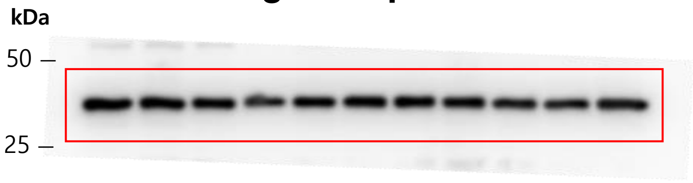

S6A Fig_PARP

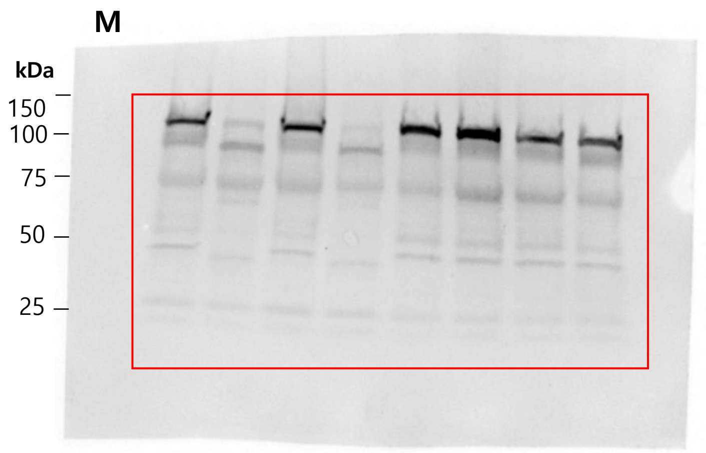

S6A Fig_C-caspase-9

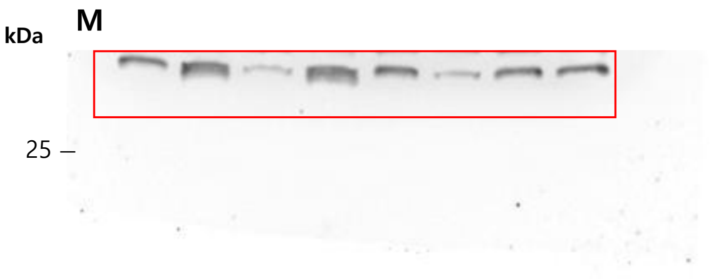

S6A Fig_C-caspase-3

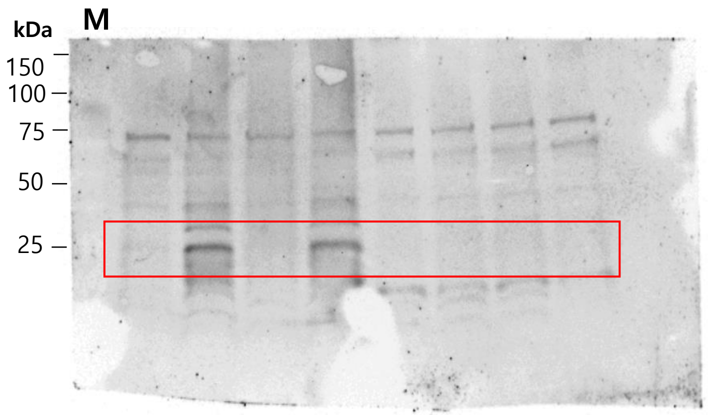

S6A Fig_Caspase-9

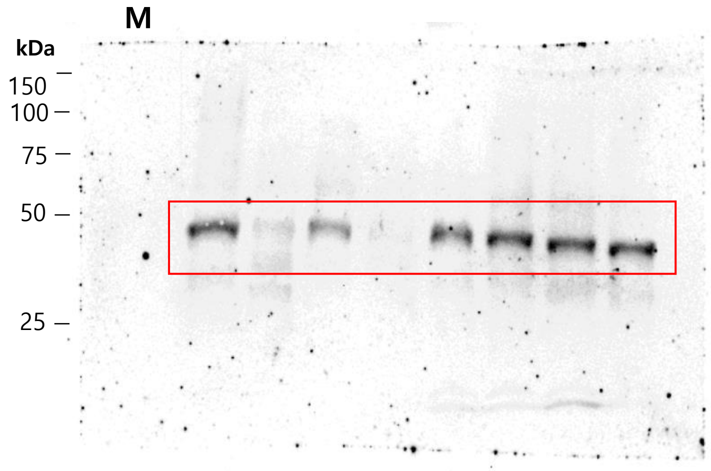

S6A Fig_Caspase-3

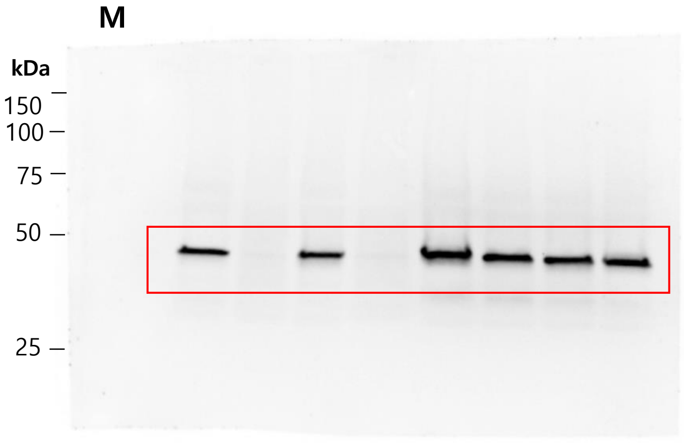

S6A Fig_β-actin

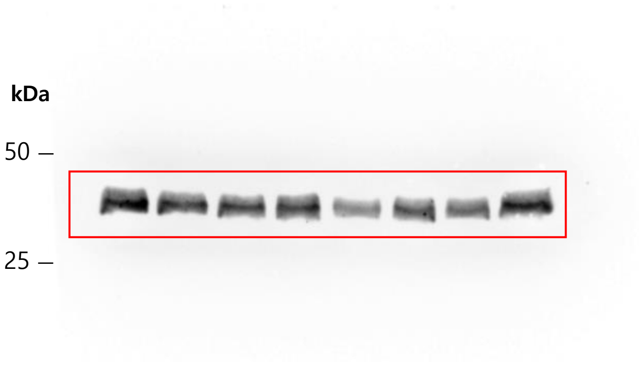

Supplement: S1 Raw Images — (PDF) [file pone.0322962.s011.pdf]
